# Effect of Early Pharmacologic Cardioversion vs. Non-early Cardioversion in the Patients With Recent-Onset Atrial Fibrillation Within 4-Week Follow-Up Period: A Systematic Review and Network Meta-Analysis

**DOI:** 10.3389/fcvm.2022.843939

**Published:** 2022-04-11

**Authors:** Yan Tang, Yujie Wang, Xuejing Sun, Yunmin Shi, Suzhen Liu, Weihong Jiang, Hong Yuan, Yao Lu, Jingjing Cai, Junru Wu

**Affiliations:** ^1^Department of Cardiology, Third Xiangya Hospital, Central South University, Changsha, China; ^2^Center of Clinical Pharmacology, Third Xiangya Hospital, Central South University, Changsha, China

**Keywords:** recent-onset, atrial fibrillation, early pharmacologic cardioversion, non-early cardioversion, network meta-analysis, randomized controlled trials

## Abstract

**Background:**

Whether early pharmacologic cardioversion is necessary for recent-onset atrial fibrillation is still controversial. Current meta-analyses were limited to evaluating the effects within 24 h without sufficient considering longer follow-up outcomes. We aimed to compare the effect of early pharmacologic cardioversion and non-early cardioversion in patients with recent-onset atrial fibrillation within 4-weeks of follow-up.

**Methods:**

We searched the Cochrane Library, EMBASE, MEDLINE, PubMed, Web of Science, ClinicalTrials.gov, and Clinicaltrialsregister. eu for randomized controlled trials (RCTs) published before November 2021 comparing early pharmacologic cardioversion and non-early cardioversion in recent-onset atrial fibrillation and synthesized data in accordance with PRISMA-Systematic Reviews and Network Meta-Analysis (NMA). Early pharmacological cardioversion referred to immediate cardioversion with antiarrhythmic drugs (i.e., amiodarone, propafenone, flecainide, tedisamil, vernakalant, vanoxerine, and sotalol) upon admission, while non-early cardioversion involved the administration of rate-control or placebo medication without immediate cardioversion.

**Results:**

16 RCTs with 2,395 patients were included. Compared to non-early cardioversion, a systematic review showed that early pharmacologic cardioversion resulted in a higher probability of sinus rhythm maintenance within 24 h (odds ratios [OR] 2.50, 95% credible interval [CrI] 1.76 to 3.54) and 1-week (2.50, 1.76 to 3.54), however, there was no significant difference in sinus rhythm maintenance within 4-weeks (1.37, 0.90 to 2.09). In subgroup analysis, the Bayesian NMA revealed that vernakalant may be successful in sinus rhythm maintenance within both 24 h (3.55, 2.28 to 5.55) and 1-week (2.72, 1.72 to 4.31). The results were consistent with the frequentist NMA.

**Conclusions:**

Non-early pharmacologic cardioversion may not be inferior to early cardioversion within a 4-week follow-up period in patients with recent-onset atrial fibrillation. The evidence remains insufficient to determine which antiarrhythmic agent is optimal in the longer run. Further high-quality relevant RCTs are necessary.

**Clinical Trial Registration:**

PROSPERO CRD42020166862.

## Introduction

The global prevalence of recent-onset atrial fibrillation accounts for nearly 26% of all types of atrial fibrillation, with substantial morbidity and mortality (1). Recent-onset atrial fibrillation is considered to be a first-detected episode of atrial fibrillation lasting no more than 7 days (2). Patients with this condition commonly receive rapid reversion to sinus rhythm by cardioversion (3–9). However, it has been a long debate over whether immediate achievement of a return to sinus rhythm is vital as recent-onset atrial fibrillation usually terminates spontaneously and pharmacologic or electrical cardioversion may result in unwanted side effects (3).

There are several randomized controlled trials (RCTs), which have proved early pharmacological cardioversion to be superior with a higher probability of cardioversion to sinus rhythm than non-early cardioversion (10–21). Among them, early pharmacological cardioversion was the immediate restoration of sinus rhythm with antiarrhythmic drugs upon admission, and non-early cardioversion included initial treatment with rate-control or placebo in the absence of early cardioversion. However, recent studies found that the proportion of patients restoring sinus rhythm at more than 7 days after early cardioversion was similar to that of patients treated with non-early cardioversion (16–19). Furthermore, a recent RACE 7 ACWAS study has also demonstrated that the non-early cardioversion was non-inferior to early cardioversion in the restoration of sinus rhythm at the prospective setting (22). For recent-onset atrial fibrillation, the current two meta-analyses compared pharmacologic cardioversion with non-cardioversion were only within a 24 h observational time frame (23, 24). However, the drug-related adverse event and cardioversion durability usually required a longer observational period to detect (12, 20, 23–26). Besides, 2021 CCS/CHRS and ESC guidelines also recommended the longitudinal management of patients with atrial fibrillation (27). Despite these, whether early pharmacologic cardioversion is superior to non-cardioversion in the sinus rhythm maintenance in the longer follow-up period remains controversial.

The success rates of cardioversion and progression of atrial fibrillation are associated with the severity of structural heart diseases and cardiovascular risk factors, are raised by guidelines and clinical trials 2018 ESC/ESH and 2020 ESC guideline (11, 12, 28–31). Nevertheless, whether early pharmacologic cardioversion is recommended in recent-onset atrial fibrillation patients with cardiovascular risk factors and structural heart diseases in the longer observational frame continues to be a matter of debate.

Therefore, we performed this systematic review and network meta-analysis to compare the efficacy of early pharmacologic cardioversion with non-early cardioversion on the restoration of sinus rhythm for up to 4-week duration based on the direct and indirect evidence. Meanwhile, we focused on both the general population of atrial fibrillation and subgroups (e.g., patients with cardiovascular risk factors and mild/moderate structural heart diseases) to increase the universality of the conclusions.

## Materials and Methods

The detailed protocol is documented online in the PROSPERO registry (CRD42020166862). This NMA was performed in accordance with the Preferred Reporting Items for Systematic Reviews and Meta-Analysis (PRISMA) statement.

### Search Strategy and Selection Criteria

We searched the Cochrane Library, EMBASE, MEDLINE, PubMed, Web of Science, ClinicalTrials.gov, and Clinicaltrialsregister. eu for randomized controlled trials (RCTs) using the MeSH search terms including atrial fibrillation and cardioversion from inception to November 2021. The MeSH search terms were atrial fibrillation and cardioversion. No language restrictions were applied. The included studies were limited by the human subject. Due to being lack of study comparing the effect of electrical cardioversion with non-early cardioversion, only trials comparing pharmacologic cardioversion with non-early cardioversion were included in our analysis. The search strategy is shown in Supplementary Systematic Review Searching Record.

We included the RCTs according to the following criteria (PICO): studies were included if they enrolled adult **patients** with atrial fibrillation of onset between 3 h and 7 days prior to admission; the **interventions** were early pharmacological cardioversion and non-early cardioversion (early pharmacological cardioversion was immediate cardioversion with antiarrhythmic drugs; non-early cardioversion was the administration of rate-control or placebo medication or without immediate cardioversion.); **comparisons** were made between early pharmacological cardioversion and non-early cardioversion; extractable **outcomes** included efficacy, safety, and prognostic endpoints. The efficacy outcomes included cardioversion to sinus rhythm within 24 h, sinus rhythm maintenance within 1-week, and sinus rhythm maintenance between 1- and 4-weeks. The safety endpoints included bradycardia, tachyarrhythmia, hypotension, gastrointestinal disorders including vomiting, nausea, diarrhea, and other digestive side effects, and nervous system disorders including hot flushes, dizziness, headache, dysgeusia, paraesthesia, and other nervous system side effects. The prognostic endpoints included all-cause mortality, stroke or transient ischemic attacks (TIA), and heart failure. The definitions of these endpoints were presented in Table S1.

### Data Extraction

Independently, two reviewers (YT, YW) screened the abstracts. For potentially eligible trials, they also assessed the full text and extracted the data. The disagreements were resolved by discussion.

### Risk of Bias Assessment

The risk of bias of the included studies was evaluated through the Cochrane Collaboration's tool (32). The risk of bias was assessed according to seven domains including allocation concealment, blinding of outcome assessment, blinding of participants and personnel, incompleteness of outcome data, randomization of sequence generation, selective outcome reporting, and “other” bias. These were rated as “low risk,” “unclear risk,” and “high risk.” And we also inspected the comparison-adjusted funnel plots of standard errors vs. effect estimates for small-study effects and publication bias only if the number of included studies with a specified endpoint was no <10 (33, 34).

### Data Synthesis and Statistical Analysis

We present the results as odds ratios (ORs) with 95% credible interval [CrI] for the Bayesian framework and with 95% confidence intervals (CIs) for the frequentist framework to summarize the statistics to quantify the efficacy, safety, and prognosis of different antiarrhythmic drugs as early pharmacologic cardioversion and non-early cardioversion. An OR greater than one represented efficacy endpoints, i.e., making early pharmacologic cardioversion better. An OR greater than one represented safety or prognostic endpoints, i.e., making non-early cardioversion better. Two-sided *p* < 0.05 was considered to be statistically significant.

The statistical heterogeneity across the included studies was evaluated as the *I*^2^ statistic. *I*^2^ > 50% was considered to be the substantial heterogeneity.

To confirm that non-early pharmacologic cardioversion may not be inferior to early cardioversion within a 4-week follow-up period in patients with recent-onset atrial fibrillation, we performed subgroup analyses as followings: (i) patients with mild/moderate structural heart disease as compared to those without structural heart disease; (ii) patients with hypertension as compared to those without hypertension; (iii) patients with continuing cardioversion for more than 24 h after immediate cardioversion as compared to those without continuing cardioversion; (iv) patients with intravenous cardioversion as compared to those without oral cardioversion. We also used *P*-values for interaction to evaluate the relationship between subgroups and treatment effect.

To further evaluate the effects of different antiarrhythmic drugs as early pharmacologic cardioversion through the direct and indirect evidence, we performed the subgroup analyses by NMA under the Bayesian framework using the pcnetmeta package (version 2.6) in R (version 3.6.1). Due to only one trial (42 participants) comparing tedisamil to non-early cardioversion, the trial was ruled out from subgroup analyses. To present the results of all the endpoints clearly, we also performed probabilistic analysis according to the cumulative rankogram (35).

Sensitivity analyses were conducted as follows: (i) The NMA under the frequentist framework using netmeta (version 1.2-0) to depict the network geometry completely. (ii) The pairwise meta-analysis with the random-effects model of DerSimonian and Laird's method using the metafor (version 2.1.0) to further provide the direct estimates across studies.

To assess the heterogeneity across the network and direct comparisons, we used the between-studies variance τ^2^ with 0.04, 0.16, and 0.36 to be a low, moderate, and high degree of heterogeneity, respectively (36). JASP (http://www.jasp-stats.org) is used to statistically evaluate inconsistencies between direct and indirect evidence globally by fitting the inconsistency model and locally estimating in closed loops (37).

To further confirm the robustness of our findings, we used three Markov chains traced with 70,000 simulated draws after a burn-in of 50,000 iterations to calculate ORs and 95% CIs using the GeMTC package (version 0.8.2). The convergence was evaluated by the trace plots and Brooks-Gelman-Rubin statistic. Model fit was measured as the total residual deviance (38).

### Quality of Evidence and Confidence in the Point Estimate

To assess the confidence in all the endpoints estimates, we used the online CINeMA (http://cinema.ispm.ch) under the GRADE (Grading of Recommendations Assessment, Development, and Evaluation) framework (39). The network estimate of each outcome was according to the six CINeMA domains including within-study bias (risk of bias in the included studies), across-study bias (publication and reporting bias), indirectness, imprecision, heterogeneity, and incoherence (differences between direct and indirect evidence) (40). The quality of the evidence in the points estimated was rated as high, moderate and low, and very low.

## Results

### Included Studies

**Figure 1:**
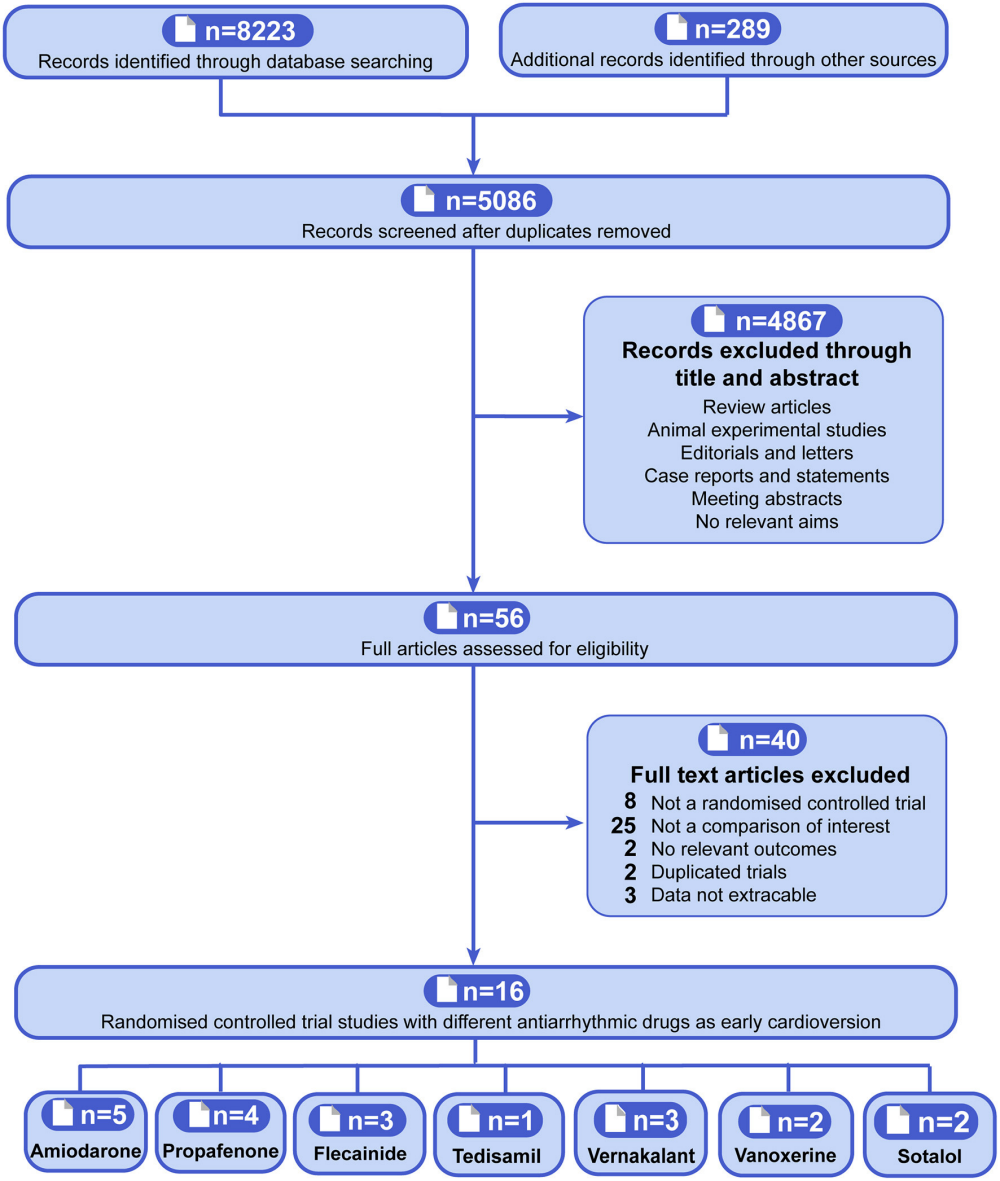
Flowchart showing the procedure for identifying the relevant publications. The antiarrhythmic drugs as early pharmacologic cardioversion in the study included amiodarone, propafenone, flecainide, tedisamil, vernakalant, vanoxerine, and sotalol.

There are overall of 5,086 citations after duplicates removed according to the included criteria, and the full-text of 56 potentially eligible articles was screened. Finally, 16 RCTs that included 2,395 participants (range of 45–437 across studies) were identified (Figure 1) (10–22, 31, 41, 42). Overall, five trials compared early pharmacologic cardioversion with amiodarone to non-early cardioversion (13, 16, 18, 20, 41), four trials compared propafenone to non-early cardioversion (10, 13–15), three trials compared flecainide to non-early cardioversion (13, 21, 22), one trial compared tedisamil to non-early cardioversion (17), three trials compared vernakalant with non-early cardioversion (11, 12, 31), two trials compared vanoxerine to non-early cardioversion (19, 42), two trials compared sotalol to non-early cardioversion (18, 20). All participants were randomly assigned 204 to amiodarone, 321 to propafenone, 310 to flecainide, 42 to tedisamil, 218 to vernakalant, 51 to vanoxerine, 85 to sotalol, and 971 to non-early cardioversion.

### Risk of Bias Within Individual Studies

The characteristics and risk of bias assessment for the included RCTs were performed and summarized (Table 1). Most studies had the lowest risks of bias for random sequence generation (16/16, 100%), selective reporting (13/16, 81.25%), incomplete outcome data (12/16, 75%), blinding of participants and personnel (13/16, 81.25%), blinding of outcome assessment (8/16, 50%), allocation concealment (14/16, 87.5%) and other bias (9/16, 56.25%). Few studies had the highest in the highest categories for blinding of outcome assessment (5/16, 31.25%) and other bias (4/16, 25%). Few studies were judged to be the highest risks of bias for unclear risk for selective reporting (3/16, 18.75%), blinding of participants and personnel (3/16, 18.75%), incomplete outcome data (4/16, 25%), other bias (3/16, 18.75%) and blinding of outcome assessment (3/16, 18.75%). Any disagreements were resolved by consensus.

**Table 1:** The characteristics and quality assessment in terms of Cochrane risk of bias assessment of included RCTs.

**Trail**	**No. of patients**	**Study design**	**Length of follow-up**	**Groups**	**Cardioversion Regimen**	**Male (%)**	**Mean age, years**	**Structural Heart Disease (%)**	**Heart Failure (%)**	**Hypertension (%)**	**Diabetes (%)**	**Valvular Heart Diseases (%)**	**Vascular Diseases (%)**	**Mean duration of Current atrial fibrillation episode, h**	**Random sequence generation**	**Allocation concealment**	**Blinding of participants and personnel**	**Blinding of outcome assessment**	**Incomplete outcome data**	**Selective reporting**	**Other bias**
Kanou pakis et al. (16)	45	Randomi zed, blind, single center	24 h	Early Pharmacologic Cardioversion	Amio 300 mg iv+Continuous iv+po	12 (52.2)	64	7 (30.4)	NA	6 (26.1)	NA	2 (8.7)	7 (30.4)	13	**+**	**+**	**?**	**?**	**+**	**+**	**–**
				Non-Early Cardioversion	Plac	9 (40.9)	65	5 (22.7)	NA	7 (31.8)	NA	2 (9.1)	6 (27.3)	11							
Boriani et al. (10)	126	Randomi zed, single-blind, multi-center	8 h	Early Pharmacologic Cardioversion	Prop 600 mg/d, po+Continuous, po	33 (51.6)	69	16 (25.0)	16 (25.0)	26 (40.6)	NA	2 (3.1)	10 (15.6)	32	**+**	**+**	**+**	**–**	**+**	**+**	**+**
				Non-Early Cardioversion	Plac	31 (43)	68	17 (27.4)	15 (24.2)	24 (38.7)	NA	6 (9.7)	7 (11.3)	31							
Donovan et al. (21)	102	Randomi zed, double-blind, single center	6 h	Early Pharmacologic Cardioversion	Flec (2 mg/kg), iv	36 (70.6)	61	NA	NA	NA	NA	NA	NA	8.7	**+**	**+**	**+**	**+**	**+**	**?**	**–**
				Non-Early Cardioversion	Plac	36 (70.6)	59	NA	NA	NA	NA	NA	NA	7.3							
Hohnloser et al. (17)	175	Randomi zed, double-blind, multi-center	28 d	Early Pharmacologic Cardioversion	Tedi (0.4–0.6 mg/kg), iv	31 (58.0)	63.7	NA	NA	NA	NA	NA	NA	25.0	**+**	**+**	**+**	**+**	**+**	**+**	**+**
				Non-Early Cardioversion	Plac	34 (58.0)	65.0	NA	NA	NA	NA	NA	NA	25.7							
Roy et al. (11)	56	Randomi zed, double-blind, multi-center	7 d	Early Pharmacologic Cardioversion	Vern 0.5–3.0 mg/kg iv and/or repeated 24 h	20 (55.6)	67.4	NA	NA	23 (63.9)	8 (22.2)	NA	NA	11.5	**+**	**+**	**+**	**+**	**+**	**+**	**?**
				Non-Early Cardioversion	Plac	14 (70.0)	64.0	NA	NA	9 (45.0)	5 (25.0)	NA	NA	13.3							
Dittrich et al. (42)	104	Randomi zed, double-blind, multi-center	7 d	Early Pharmacologic Cardioversion	Vano 400 mg/d, po	12 (48.0)	68.4	13 (40.6)	4 (16.0)	41 (56.9)	2 (3.0)	6 (24.0)	1 (4.0)	64.8	**+**	**+**	**+**	**+**	**+**	**+**	**+**
				Non-Early Cardioversion	Plac	22 (68.8)	62.3	31 (43.1)	10 (31.3)	22 (68.8)	0 (0.0)	4 (12.5)	0 (0.0)	33.6							
Thomas et al. (20)	140	Randomi zed, blind, single center	12 h	Early Pharmacologic Cardioversion	Amio 10 mg/kg, iv+Continuous, po	19 (36.5)	54.3	9 (17.3)	0	15 (28.8)	NA	2 (3.8)	NA	NA	**+**	**?**	**?**	**?**	**–**	**?**	**?**
				Early Pharmacologic Cardioversion	Sota 1.5 mg/kg, iv+Continuous, po	5 (11.1)	57.7	6 (13.0)	0	14 (31.1)	NA	2 (4.4)	NA	NA							
				Non-Early Cardioversion	Plac	20 (46.5)	55.5	11 (25.6)	0	8 (18.6)	NA	7 (16.3)	NA	NA							
Galve et al. (41)	100	Randomized, single-blind, single center	15 d	Early Pharmacologic Cardioversion	Amio 5 mg/kg iv+Continuous 24 h	27 (54.0)	60	22 (44.0)	5 (10.0)	NA	NA	NA	NA	25	**+**	**+**	**+**	**–**	**+**	**+**	**?**
				Non-Early Cardioversion	Plac	28 (56.0)	61	30 (60.0)	6 (12.0)	NA	NA	NA	NA	18							
Roy et al. (12)	220	Randomized, double-blind, multi-center	30 d	Early Pharmacologic Cardioversion	Vern 3 mg/kg iv and/or repeated	48 (64.0)	60.4	34 (23.4)	14 (10.0)	57 (39.0)	10 (7)	NA	NA	28.2	**+**	**+**	**+**	**+**	**–**	**+**	**–**
				Non-Early Cardioversion	Plac	102 (70.3)	59.9	17 (22.7)	5 (7.0)	32 (43.0)	4 (5)	NA	NA	28.4							
Bellandi et al. (14)	182	Randomized, single-blind, single center	24 h	Early Pharmacologic Cardioversion	Prop 2 mg/kg iv+Continuous 24 h	NA	65.2	50 (51.0)	NA	19 (19.3)	NA	20 (20.4)	24 (24.4)	57.0	**+**	**+**	**+**	**–**	**–**	**?**	**+**
				Non-Early Cardioversion	Plac	NA	66.1	44 (52.4)	NA	19 (22.6)	NA	17 (20.2)	19 (22.6)	49.8							
Boriani et al. (15)	240	Randomized, single-blind, multi-center	8 h	Early Pharmacologic Cardioversion	Prop 600 mg/d, po	70 (58.8)	59	32 (26.9)	27 (22.7)	37 (31.1)	NA	8 (6.7)	11 (9.2)	31	**+**	**+**	**+**	**–**	**+**	**+**	**+**
				Non-Early Cardioversion	Plac	67 (55.4)	58	30 (24.8)	26 (21.5)	37 (30.6)	NA	9 (7.4)	9 (7.4)	30							
Balla et al. (13)	160	Randomized, single-blind, single center	24 h	Early Pharmacologic Cardioversion	Flec 3 mg/kg, po	28 (70.0)	57.9	NA	NA	18 (45.0)	10 (25.0)	NA	NA	16.2	**+**	**+**	**+**	**–**	**+**	**+**	**+**
				Early Pharmacologic Cardioversion	Amio 30 mg/kg, po	29 (72.5)	58.9	NA	NA	12 (30.0)	16 (40.0)	NA	NA	19.1							
				Early Pharmacologic Cardioversion	Prop 8.5 mg/kg, po	20 (50.0)	57.4	NA	NA	20 (50.0)	12 (30.0)	NA	NA	18.6							
				Non-Early Cardioversion	Plac	24 (60.0)	58.6	NA	NA	9 (22.5)	8 (20.0)	NA	NA	17.8							
Piccini et al. (19)	41	Randomized, double-blind, multi-center	30 d	Early Pharmacologic Cardioversion	Vano 400 mg/d, po	21 (79.2)	68.1	18 (69.2)	5 (10.8)	16 (66.7)	4 (16.7)	10 (41.7)	NA	NA	**+**	**+**	**+**	**+**	**+**	**+**	**–**
				Non-Early Cardioversion	Plac	8 (53.3)	66.9	8 (53.3)	2 (14.3)	11 (78.6)	0 (0.0)	4 (28.6)	NA	NA							
Joseph et al. (18)	115	Randomized, double-blind, multi-center	48 h	Early Pharmacologic Cardioversion	Amio 5 mg/kg iv+400 mg, 48 h, po	25 (64.1)	61.3	21 (53.8)	8 (20.5)	5 (8, 12)	NA	3 (7.7)	8 (20.5)	NA	**+**	**+**	**+**	**+**	**+**	**+**	**+**
				Early Pharmacologic Cardioversion	Sota 1.5 mg/kg iv+80 mg, 48 h, po	19 (47.5)	62.8	14 (35.0)	6 (15.0)	6 (15.0)	NA	1 (2.50)	7 (17.5)	NA							
				Non-Early Cardioversion	Plac	20 (55.6)	64.9	18 (50.0)	6 (16.7)	10 (27.8)	NA	4 (11.1)	3 (8.3)	NA							
Pluymaekers et al. (22)	437	Randomized, open-label, multi-center	28 d	Early Pharmacologic Cardioversion	Flec 2 mg/kg, iv	130 (59.0)	65	13 (5.94)	NA	133 (61.0)	25 (11.0)	NA	13 (6.0)	NA	**+**	**–**	**?**	**?**	**+**	**+**	**+**
				Non-Early Cardioversion	Plac	131 (60.0)	65	24 (11.0)	NA	118 (54.0)	21 (10.0)	NA	24 (11.0)	NA							
Beatch et al. (31)	111	Randomized, double-blind, multi-center	30 d	Early Pharmacologic Cardioversion	Vern 3 mg/kg iv and/or repeated 24 h	37 (67.0)	60.7	11 (20.0)	5 (9.0)	NA	NA	12 (20.0)	NA	48	**+**	**+**	**+**	**+**	**–**	**+**	**+**
				Non-Early Cardioversion	Plac	30 (54.0)	59.2	13 (23.0)	3 (5.0)	NA	NA	13 (23.0)	NA	48							

*iv, intravenous; po, oral; TIA, Transient Ischemic Attack; Amio, Amiodarone; Sota, Sotalol; Vern, Vernakalant; Vano, Vanoxerine; Prop, Propafenone; Flec, Flecainide; Tedi, Tedisamil; Plac, Placebo; NA, not available. 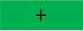 Low Risk; 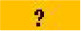 Unclear Risk; 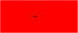 High Risk*.

### Outcomes of the Network Meta-Analysis and Quality Assessments of Endpoints

#### Efficacy Results

**Figure 2:**
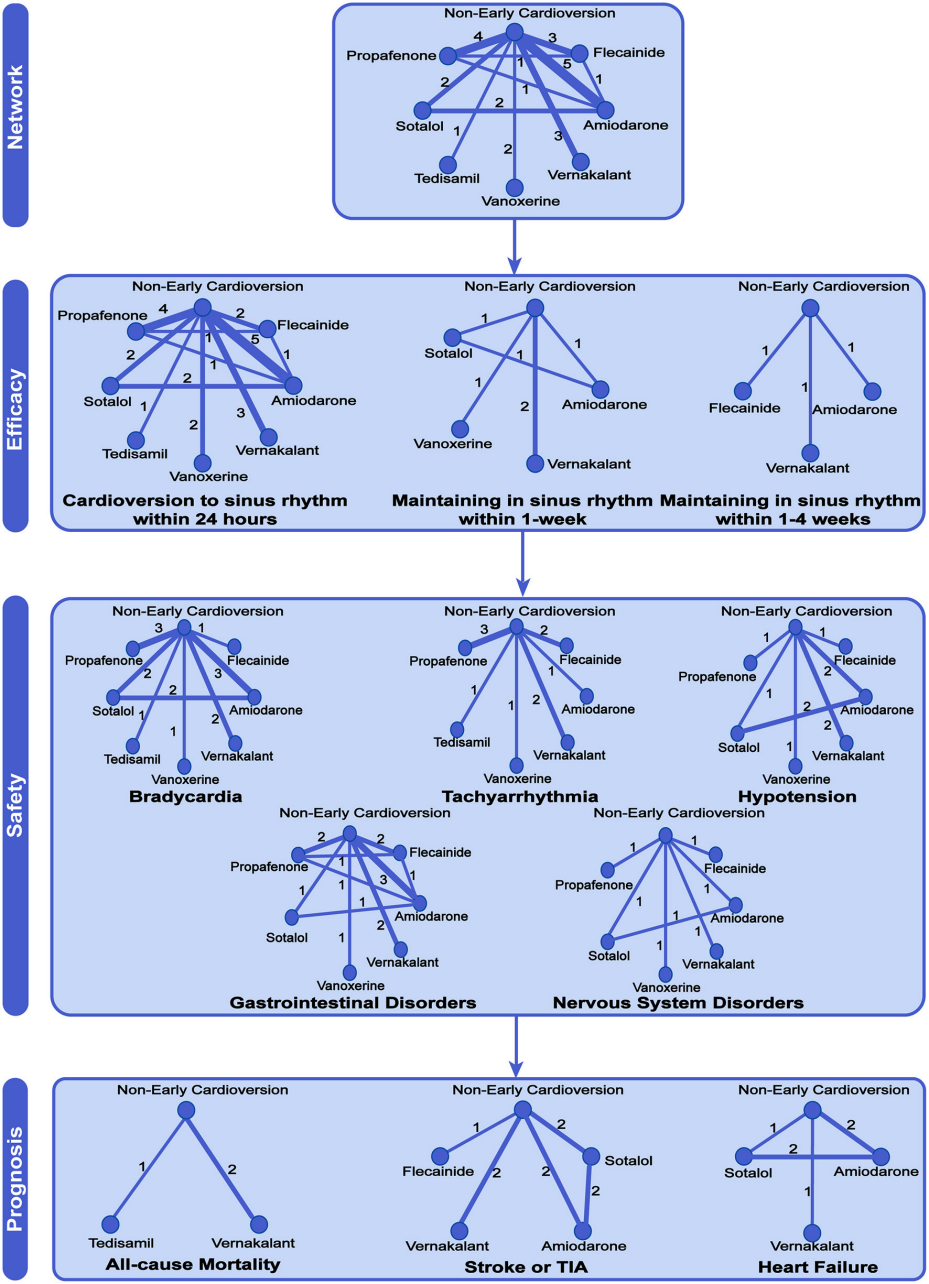
Network meta-analysis of available comparisons. Circular nodes show different antiarrhythmic drugs as early pharmacologic cardioversion and non-early cardioversion. The antiarrhythmic drugs in the study included amiodarone, propafenone, flecainide, tedisamil, vernakalant, vanoxerine, and sotalol. Line width is proportional to the number of trials including each pair of treatments (direct comparisons). TIA, Transient Ischemic Attack.

Fifteen studies with 1,958 participants reported the rate of cardioversion to sinus rhythm within 24 h, 4 studies containing 501 participants reported the rate of maintaining sinus rhythm within 1-week and 3 studies with 644 participants reported the rate of maintaining sinus rhythm between 1- and 4-weeks, as shown in Figure 2.

**Figure 3:**
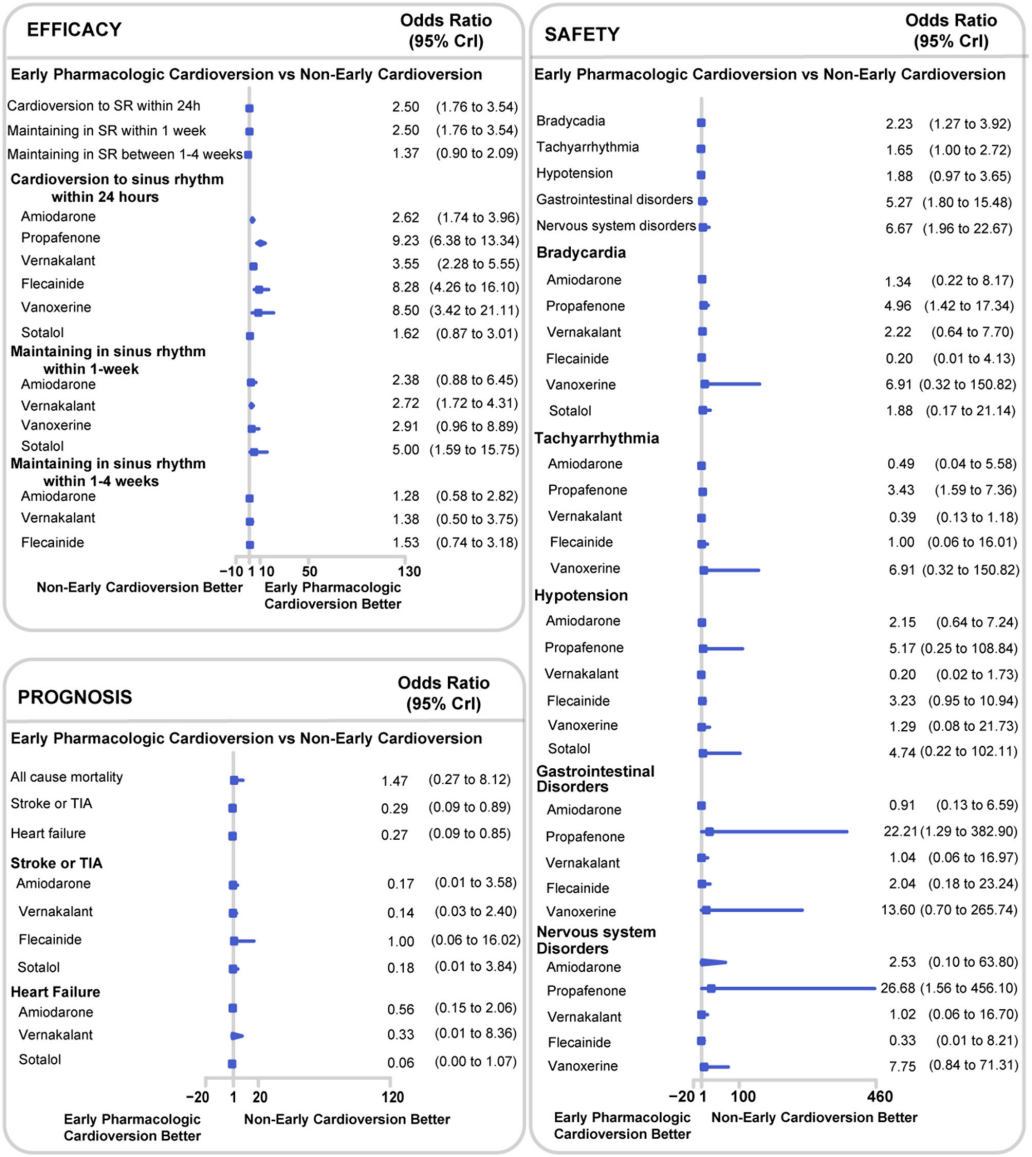
Network meta-analysis results for all endpoints between early pharmacologic cardioversion and non-early cardioversion. The forest plot was based on the Bayesian framework. The antiarrhythmic drugs as early pharmacologic cardioversion in the study included amiodarone, propafenone, flecainide, tedisamil, vernakalant, vanoxerine, and sotalol. All endpoints are efficacy endpoints including cardioversion to sinus rhythm within 24 h, maintenance in sinus rhythm within 1-week and maintenance in sinus rhythm within 1–4-weeks, safety endpoints including bradycardia, tachyarrhythmia, hypotension, gastrointestinal disorders, and nervous system disorders, and prognostic endpoints including all-cause mortality, stroke or TIA and heart failure. CrI, Credible Interval; TIA, Transient Ischemic Attack.

The early pharmacologic cardioversion was more effective in cardioversion to sinus rhythm within 24 h (OR 2.50, 95% CrI 1.76 to 3.54, *I*^2^ = 0.82, *p* < 0.0001) and maintaining sinus rhythm within 1-week than non-early cardioversion (2.50, 1.76 to 3.54, *I*^2^ = 0.79, *p* = 0.01) under the Bayesian framework, as presented in Figure 3. These results indicated that the efficacy of cardioversion to sinus rhythm within 24 h and maintaining sinus rhythm within 1-week might be significantly increased when the initiation of cardioversion earlier. However, maintaining sinus rhythm between 1 and 4-weeks showed no significant differences (1.37, 0.90 to 2.09, *I*^2^ = 0.00, *p* = 0.95). According to CINeMA, the quality of the efficacy outcomes of maintaining sinus rhythm within 4-week was determined to be low and moderate mainly due to imprecision and heterogeneity, as shown in Table S2.

#### Safety Results

There were 11 studies with 1,579 participants reported the rate of bradycardia, 10 studies with 1,481 participants reported the rate of tachyarrhythmia, 8 studies with 903 participants reported the rate of hypotension, 8 studies with 850 participants reported the rate of gastrointestinal disorders, and 5 studies with 592 participants reported the rate of nervous system disorders (Figure 2). Compared with non-early cardioversion, early pharmacologic cardioversion significantly increased the side effects of bradyarrhythmia (OR 2.23, 95%CrI 1.27 to 3.92, *I*^2^ = 0.00, *p* = 0.30), gastrointestinal disorders (5.27, 1.80 to 15.48, *I*^2^ = 0.14, *p* = 0.07) and nervous system disorders (6.67,1.96 to 22.67, *I*^2^ = 0.29, *p* = 0.12), but not tachycardia (1.65, 1.00 to 2.72, *I*^2^ = 0.36 *p* = 0.47) and hypotension (1.88, 0.97 to 3.65, *I*^2^ = 0.10, *p* = 0.19) under the Bayesian framework (Figure 3). The quality of these safety outcomes was rated to be low due to imprecision, incoherence, and heterogeneity through the CINeMA approach, as presented in Table S2.

#### Prognostic Results

There were 3 studies with 419 participants reported the rate of all-cause mortality, 5 studies with 841 participants reported the rate of stroke or TIA, and 3 studies with 366 participants reported the rate of heart failure (Figure 2).

The early pharmacologic cardioversion in comparison with non-early cardioversion decreased the risk of stroke or TIA (0.29, 0.09 to 0.89, *I*^2^ = 0.00, *p* = 0.08) and heart failure (0.27, 0.09 to 0.85, *I*^2^ = 0.27, *p* = 0.17), however, early pharmacologic cardioversion did not significantly decrease the risk of all-cause mortality (1.47, 0.27 to 8.12, *I*^2^ = 0.06, *p* = 0.76) under the Bayesian framework (Figure 3). Using CINeMA, the serious across-study bias, imprecision, incoherence, and heterogeneity were resulted in the low quality of these prognostic outcomes, as shown in Table S2.

### Subgroup Analyses Based on Different Antiarrhythmic Drugs for the Early Pharmacologic Cardioversion

**Figure 4:**
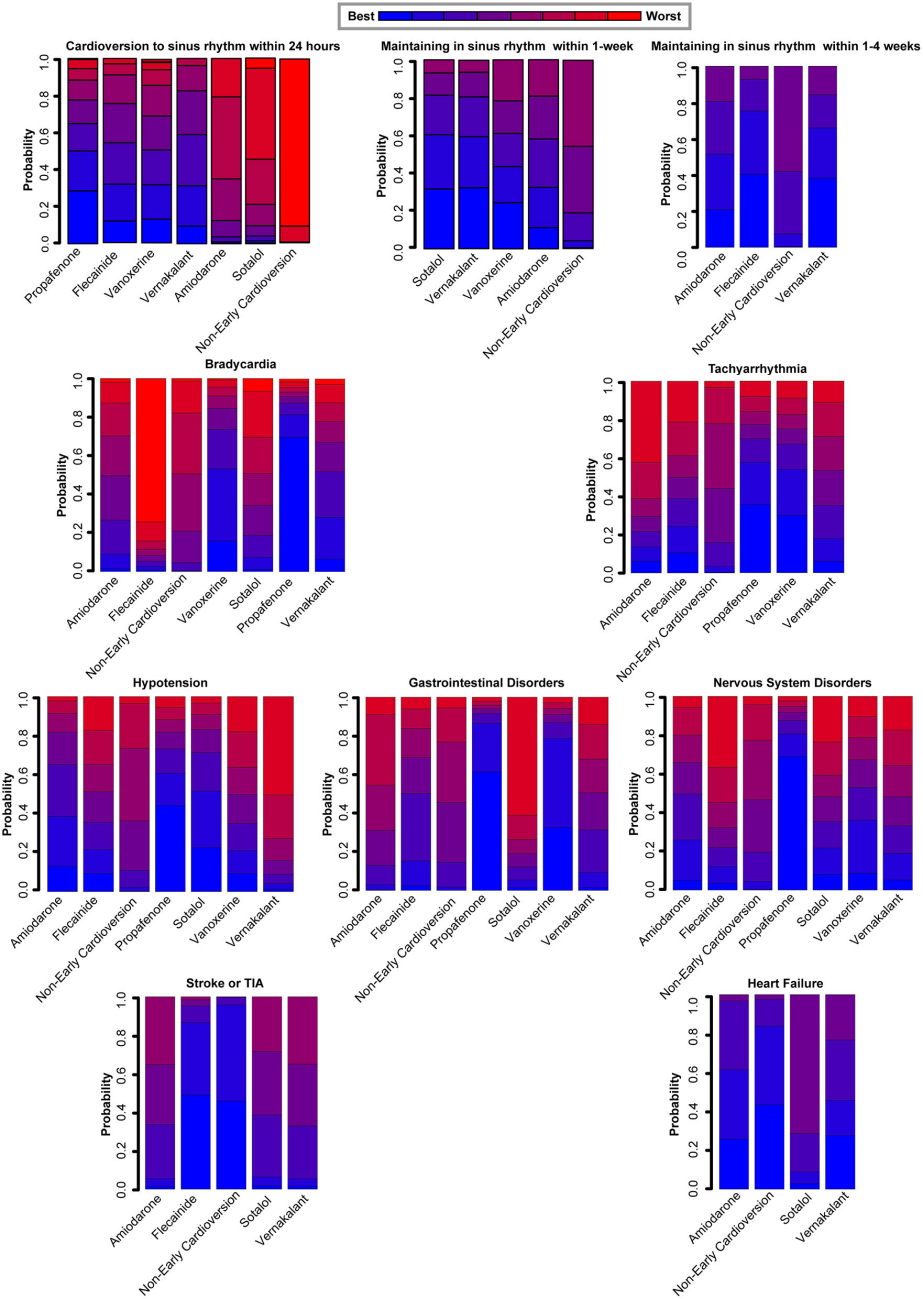
Rankograms demonstrate the probability of each therapy ranked across all endpoints. ^*^The antiarrhythmic drugs as early pharmacologic cardioversion in the study included amiodarone, propafenone, flecainide, vernakalant, vanoxerine, and sotalol.^†^All endpoints are efficacy endpoints including cardioversion to sinus rhythm within 24 h, maintenance in sinus rhythm within 1-week and maintenance in sinus rhythm within 1–4-weeks, safety endpoints including bradycardia, tachyarrhythmia, hypotension, gastrointestinal disorders, and nervous system disorders, and prognostic endpoints including stroke or TIA and heart failure. TIA, Transient Ischemic Attack.

The subgroup analyses on different antiarrhythmic drugs showed early pharmacologic cardioversion yielded consistent results with the primary analysis, as exhibited in Figure 3. Early pharmacologic cardioversion, exception of sotalol (1.62, 0.87 to 3.01), may lead to a higher probability of a return to sinus rhythm within 24 h than non-early cardioversion. However, sotalol (5.00, 1.59 to 15.75) may achieve a greater chance of maintaining sinus rhythm at 1-week. Vernakalant may be successful in sinus rhythm maintenance not only within 24 h (3.55, 2.28 to 5.55) but also in the 1-week follow-up (2.72, 1.72 to 4.31). The probabilistic analysis ranked propafenone high and sotalol low for cardioversion to sinus rhythm within 24 h, vernakalant high for sinus rhythm maintenance at 24 h and within 1-week, as shown in Figure 4. Nevertheless, we also found that included antiarrhythmic drugs as early pharmacologic cardioversion might be non-superior to non-early cardioversion in the efficacy of maintaining sinus rhythm within 1–4-weeks.

For the safety outcomes, propafenone had a higher incidence of side-effects including bradycardia (4.96, 1.42 to 17.34), tachyarrhythmia (3.43, 1.59 to 7.36), gastrointestinal disorders (22.21, 1.29 to 382.90), nervous system disorders (26.68, 1.56 to 456.10) compared to non-early cardioversion. However, there was no significant risk obtained for all safety endpoints in the other antiarrhythmic agents. Meanwhile, probabilistic analysis ranked propafenone high for the adverse effects during 4-weeks follow-up, as presented in Figure 4.

For the prognostic outcomes, non-early cardioversion might be non-inferior to these mentioned-above antiarrhythmic drugs as early pharmacologic cardioversion. However, serious across-study bias, imprecision, and heterogeneity existed in these endpoints estimates of subgroup analyses. Consequently, there is insufficient evidence to determine which antiarrhythmic drug is superior, only providing a degree of confidence in the interpretation of results.

### Sensitivity Analyses

The efficacy, safety, and prognostic results of the prespecified sensitivity analyses from NMA under the frequentist framework and standard pairwise direct meta-analyses, as shown in Figures S1, S2 were not significantly different from those from NMA under the Bayesian framework.

### Network Coherence and Quality of Evidence

There were no obvious discrepancies between direct and indirect estimates in closed loops that allowed the evaluation of network coherence for all the outcomes other than stroke or TIA, as presented in Table S3. The total residual deviance for all the endpoints, except for gastrointestinal disorders and stroke or TIA, as revealed in Table S4, suggested a good consistent model fit under the Bayesian framework.

### Publication Bias and Small-Study Effects

Funnel plots for endpoints with more than 10 studies including cardioversion to sinus rhythm within 24 h, bradycardia; gastrointestinal disorders, and hypotension showed that there was no significant evidence for publication bias, as suggested in Figure S3. However, the other endpoints with <10 studies (i.e., maintaining in sinus rhythm within 1-week; maintaining in sinus rhythm within 1–4-weeks; tachyarrhythmia; hypotension; nervous system disorders; all-cause mortality; stroke or TIA; heart failure) were needed to downgrade one level for risk of bias.

### Additional Subgroup Analyses

**Figure 5:**
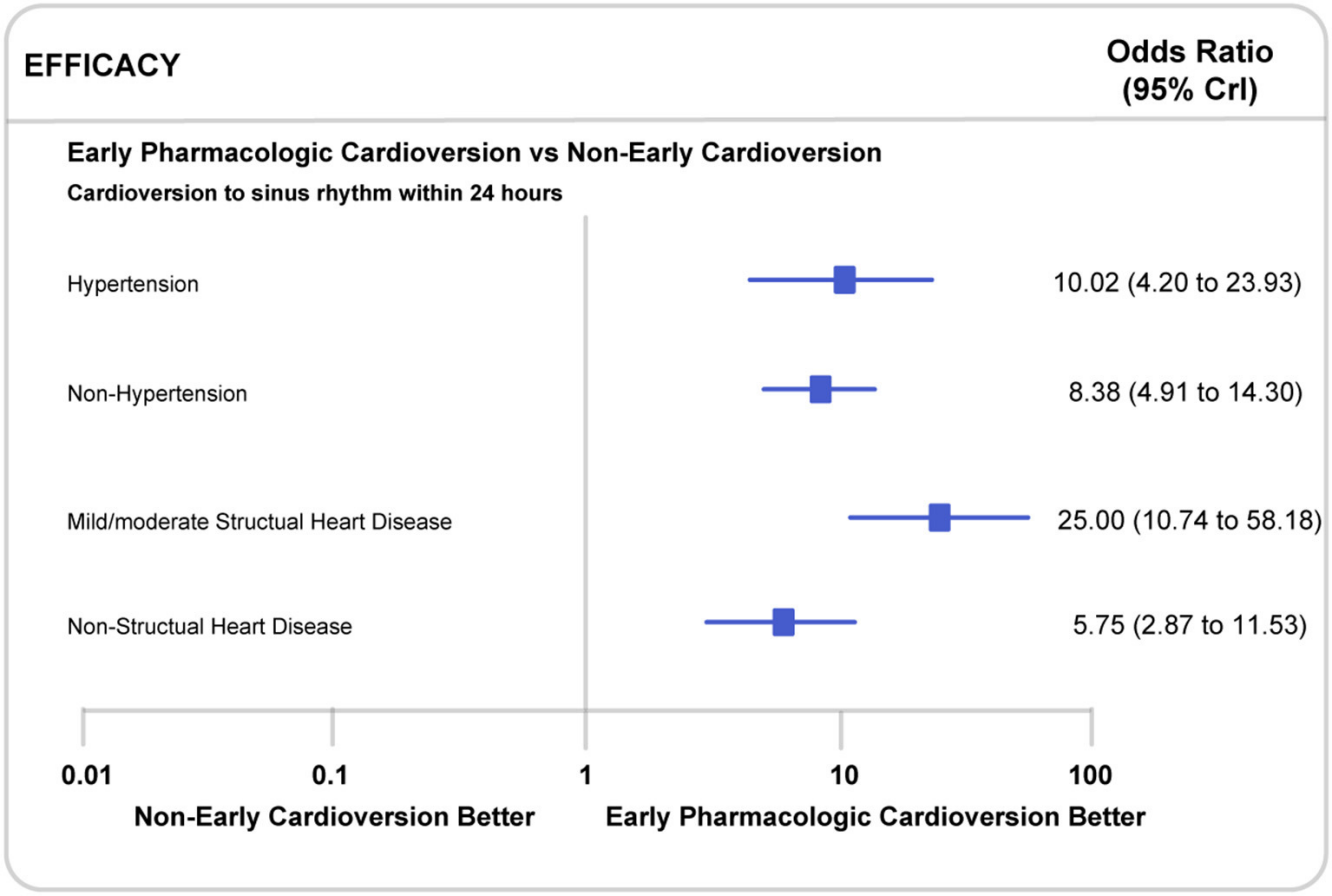
Subgroup analyses in recent-onset atrial fibrillation patients with or without mild/moderate structural heart disease or hypertension. CrI, Credible Interval.

Propafenone might be more effective in restoring sinus rhythm within 24 h in the subgroup with mild/moderate structural heart disease (25.00, 10.74 to 58.18, P for interaction <0.0001) than in patients without structural heart disease (5.75, 2.87 to 11.53, P for interaction <0.0001). Similarly, propafenone showed better performance in the patients with hypertension (10.02, 4.20 to 23.93, P for interaction <0.0001) than those without (8.38, 4.91 to 14.30, P for interaction <0.0001) (Figure 5). Considering the impact of the exposure time and route of all included antiarrhythmic drugs, the additional subgroup analyses indicated that there was no significant difference in cardioversion to sinus rhythm within 24 h whether continuing (2.63, 1.76 to 3.93, P for interaction <0.0001) or non-continuing cardioversion (4.61, 3.68 to 5.79, P for interaction <0.0001). Besides, no substantial difference was observed in the restoration of sinus rhythm within 1-week. Also, in subgroups of patients with oral (7.16, 5.07 to 10.11, P for interaction <0.0001) or intravenous (2.99, 2.35 to 3.81, P for interaction <0.0001) treatment, we did not observe a noticeable difference between early pharmacologic cardioversion and non-early pharmacologic cardioversion for sinus rhythm maintenance within 24 h (Figure S4). However, due to the lack of this study-level subgroup data on long-term efficacy and safety endpoints, we were unable to comprehensively assess the superiority of early pharmacologic cardioversion. The head-to-head trial comparing in this large patient population is warranted in the future.

## Discussion

There are six novel discoveries from our study. **First**, the analysis showed that early pharmacologic cardioversion resulted in a higher rate of cardioversion to sinus rhythm within 24 h and sinus rhythm maintenance within 1-week than the non-cardioversion. However, early cardioversion might not benefit from maintaining sinus rhythm in 1 to 4-weeks. **Second**, early pharmacologic cardioversion in the 4-week follow-up may not reduce the risk of all-cause mortality. **Third**, compared to non-early cardioversion groups, seven common anti-arrhythmic agents are beneficial for maintaining sinus rhythm within 1-week, while failing to demonstrate an effect between 1- to 4- weeks. **Fourth**, anti-arrhythmic medication led to higher risks of drug-associated side effects in the 4-week observational time window. **Fifth**, early pharmacologic cardioversion with vernakalant might be superior to other drug regimes in maintaining sinus rhythm within 1-week. In contrast, propafenone treatment showed the highest adverse effects, including bradycardia, tachyarrhythmia, gastrointestinal disorders, and nervous system disorders. **Sixth**, we found patients with mild/moderate structural heart disease or hypertension may benefit more from early pharmacologic cardioversion than those without structural heart disease or hypertension in terms of sinus rhythm maintenance within 24 h. The effect of maintaining sinus rhythm within 24 h among patients with mild/moderate structural heart disease or hypertension was only evaluated in patients with propafenone treatment. Whether other antiarrhythmic agents would be superior to propafenone remains assessed.

Current AHA/ACC/HRS, ESC, and Canadian Cardiovascular Society Guidelines recommend early pharmacologic cardioversion for recent-onset atrial fibrillation. Early pharmacologic cardioversion has been proved superior with a higher probability of cardioversion to sinus rhythm and earlier elimination of symptoms than the non-early cardioversion in 24 h' time frame in our study (29, 30, 44). Moreover, our evidence also demonstrated that patients might benefit from early pharmacologic cardioversion for a 1-week follow-up. However, the results from four RCTs and our study demonstrated that efficacy from early pharmacologic cardioversion might not prolong to 4-weeks (22, 31, 41, 42). Own to the limited number of studies beyond 24 h, the efficacy of early pharmacologic cardioversion warrants further confirmation by high-quality studies.

Previous studies considered that early cardioversion may prevent persistent atrial fibrillation and reduce the risk of heart failure and cerebral ischemic events (10, 21). However, current RCTs found that reduction of the risk of stroke is primarily counted on the timely initiation of anticoagulation in recent-onset atrial fibrillation, regardless of the early pharmacologic cardioversion (22, 29, 30, 45, 46). In our study, we found that the early pharmacologic cardioversion might not significantly decrease the risk of all-cause mortality, but may reduce the risk of stroke and heart failure, compared to the non-early cardioversion within a 4-week follow-up. However, due to the high heterogeneity of the included studies, this conclusion needs to be confirmed in a study with a rigorous design.

2020 Canadian Cardiovascular Society/Canadian Heart Rhythm Society Comprehensive Guidelines referred to the risk of progression and recurrence of atrial fibrillation increases with the severity of cardiovascular risk factors such as hypertension, diabetes mellitus, obesity, and structural heart disease. 2018 ESC/ESH, 2020 ESC guideline also raised the importance of hypertension and structural heart disease as contributors to atrial fibrillation development (28–30). Therefore, the selection of anti-arrhythmic drugs should be based on concomitant structural or functional heart disease (11, 47–50). However, there was scarce evidence of comparing efficacy and safety for early cardioversion across the concomitant structural disease in patients with recent-onset atrial fibrillation (47). In our current study, we explored the efficacy and safety of comparing early pharmacologic cardioversion with non-early cardioversion in the subgroups of patients with mild/moderate structural heart disease or hypertension to comprehensively evaluate the superiority of early pharmacologic cardioversion in the short and long duration. We observed a noticeable difference between early pharmacologic cardioversion with non-early cardioversion for efficacy at 24 h in patients with hypertension and mild/moderate structural heart disease. Spontaneous cardioversion rarely occurs in atrial fibrillation patients with structural heart disease (10). Thus, early pharmacologic cardioversion might be recommended in patients with existing cardiovascular disease.

The optimal drug for early pharmacologic cardioversion in patients with atrial fibrillation does not consistently recommend in expert consensus and guidelines (43). For example, UK guidelines recommend flecainide (43), European guidelines recommend vernakalant (9), and U.S. guidelines recommend flecainide (51). In addition to guidelines, two meta-analyses suggested that vernakalant may be an optimal option for recent-onset atrial fibrillation (52, 53). Recent network meta-analyses found that vernakalant and flecainide may be relatively more efficacious agents than other anti-arrhythmic drugs for recent-onset atrial fibrillation, albeit at different observational time windows (23, 24, 47). Our results indicated that vernakalant might have better performance for recent-onset atrial fibrillation than other included antiarrhythmic agents due to its effectiveness both in immediate cardioversion and sinus rhythm maintenance within 1-week, despite vernakalant is initially found to be the most rapidly cardioverting drug (29) due to 3–8.5 h half-life span. Our results about vernakalant are consistent with recent RCTs and meta-analysis within more than 24 h observational time window (31, 52), which might be explained by its CYP2D6 genotype. Meanwhile, we also found that the use of propafenone may be associated with significant side effects. The adverse effects are largely dose-related and are very uncommon at the doses used for atrial fibrillation (54). However, all the selected antiarrhythmic agents, including propafenone, were at the recommended and well-tolerated dose. In our meta-analysis, we are the first to extend the observational time to a 4-week follow-up to show comprehensive safety profiles for early pharmacologic cardioversion, which might help physicians determine which treatment is superior. Therefore, the adverse events summarized in our study might be partly explained by the longer observational time. Additionally, the conditions of patients who had recurrent atrial fibrillation with poor rate control or left ventricular failure (18) and its CYP2D6 genotype (25, 31, 55), might also contribute to the drug-related adverse effects based on the safe dose. Due to the lack of sufficient direct and indirect evidence among the antiarrhythmic drugs, more high-quality relevant RCTs with longer-term follow-up are essential to determine the efficacy, side-effect, and thrombotic risk of the antiarrhythmic drugs as early pharmacologic cardioversion.

The different exposure times, properties, serum-concentration time, and route of administration in antiarrhythmic agents may bring significant heterogeneity to the therapeutic effects (23, 24). Therefore, we conducted the additional sensitivity analyses under the frequentist framework and pairwise meta-analysis with the random-effects model to further provide direct comparisons (56). Compared with indirect comparisons, we found the result was not changed. Furthermore, we performed the additional subgroup analyses according to continuing cardioversion for more than 24 h after immediate cardioversion and cardioversion deliveries in patients with recent-onset atrial fibrillation, the result showed that there was no significant difference in both subgroups. Therefore, we considered the heterogeneity might not significantly affect the results. Future prospective studies with a larger population are needed to confirm this conclusion.

Our systematic review and network meta-analysis have several limitations. **First**, only three studies were comparing the efficacy of maintaining sinus rhythm in 1–4-weeks between the early pharmacologic cardioversion arm and the non-early cardioversion arm. Besides, we only obtained the efficacy, safety, and prognostic endpoints within 4-weeks due to the limited follow-up period (range: 6 h−30 days) in the included studies. Prospective studies with a larger population and extended follow-up time are warranted. **Second**, the numbers of studies comparing the efficacy of maintaining sinus rhythm between anti-arrhythmic medication are limited. High-quality randomized trials may be required to further verify the observations from our study. **Third**, there are unadjusted confounders, i.e., left atrial size and dose of anti-arrhythmic drugs, in studies potentially impact network stability and generalizability of our study. Own to the too small sample sizes in our meta-analysis, we could not adjust for potential confounders. Additional evidence warrants to conduct covariate-adjusted analysis in the future. **Fourth**, the details of the medication of anticoagulation were unclear in the present studies, led to significant heterogeneity. **Fifth**, whether the other rhythm control attempts in the non-early cardioversion group was made within 4-week follow-up period was unknown, may affect the reliability of the conclusions in our study. **Sixth**, the cost-effectiveness assessment of emergency room admission should be also considered in this context, while we did not analyze this aspect due to the data unavailability from the clinical trials. **Seventh**, this study was focused on outcomes within 4-week of follow-up due to limited follow-up duration in the included studies. **Eighth**, despite the patients with structural heart disease, hypertension, and other cardiovascular diseases enrolled in most included studies, the number of patients is not adequate to make a solid conclusion on whether early pharmacologic cardioversion would benefit these patients' subpopulations. **Ninth**, although we applied random-effects Bayesian network meta-analysis and the Cochrane Risk of Bias (RoB) Assessment Tool with the CINeMA framework, the undocumented clinical characteristics of included patients (e.g., type of structural heart disease, degree of atrial heart disease, older age, valve disease, previous ECG in sinus rhythm) may bring large heterogeneity to each study (40, 57). Further study should include more detailed clinical information to reduce potential bias. Despite these limitations, our results somewhat indicated that early pharmacologic cardioversion may not be beneficial in the long run.

## Conclusions

In patients with recent-onset atrial fibrillation, the individuals with early pharmacologic cardioversion might not be superior to those with non-early cardioversion regarding the effect of maintaining sinus rhythm within 4-weeks follow-up. While vernakalant may be the viable option, considering the efficacy of maintaining sinus rhythm in 1-week. Although we cannot draw a definite conclusion on which approach or agent is optimal, our analysis provides an intriguing hypothesis for the prospective studies. Further high-quality RCTs and head-to-head trials between antiarrhythmic drugs for a longer observational period are needed.

## Data Availability Statement

The datasets presented in this study can be found in online repositories. The names of the repository/repositories and accession number(s) can be found in the article/Supplementary Material.

## Author Contributions

YT and YW conceived and designed the project. JW and JC supervised the project. JW performed the review and approval of the manuscript. YT, YW, XS, YS, and SL contributed to the design of the study, writing the protocol, screening trials, data extraction, and analysis. YL, HY, and JW contributed to the interpretation of the results. YT and YW generated the tables and figures and drafted the manuscript. YT, YW, JC, and JW participated in the formal revision, including data processing, statistical analysis, generating figures and tables, and text modification. All authors had full access to the data in the study and can take responsibility for the integrity of the data and the accuracy of the data analysis.

## Funding

This work was supported by the National Natural Science Foundation of China [grant numbers 81870171, JC; grant numbers 81800393, YL; grant numbers 81770403, HY; grant numbers 81974054, HY]; the National Key Research and Development Projects [grant numbers 2019YFF0216300, JC; grant numbers 2018YFC1311300, WJ]; the Independent Exploration and Innovation Project for Graduate Students of Central South University [grant numbers 2020zzts297, to YT]; and the Hunan Distinguished Young Scholars [grant number 2017RS3015, JC].

## Conflict of Interest

The authors declare that the research was conducted in the absence of any commercial or financial relationships that could be construed as a potential conflict of interest.

## Publisher's Note

All claims expressed in this article are solely those of the authors and do not necessarily represent those of their affiliated organizations, or those of the publisher, the editors and the reviewers. Any product that may be evaluated in this article, or claim that may be made by its manufacturer, is not guaranteed or endorsed by the publisher.
