# Supplementary material for: Effect of Early Pharmacologic Cardioversion vs. Non-early Cardioversion in the Patients With Recent-Onset Atrial Fibrillation Within 4-Week Follow-Up Period: A Systematic Review and Network Meta-Analysis
Source: Front Cardiovasc Med. 2022 Apr 11;9:843939. doi: 10.3389/fcvm.2022.843939 (PMC9036487; doi:10.3389/fcvm.2022.843939)
Supplement: Supplementary file 1 [file Data_Sheet_1.pdf]

Effect of Early Pharmacologic Cardioversion vs Non-Early Cardioversion in the Patients with Recent-Onset Atrial Fibrillation within Four-Week Follow-Up Period: A Systematic Review and Network Meta-Analysis

Systematic Review Searching Record.

Supplemental Figure 1. Network meta-analysis results for all endpoints between early pharmacologic cardioversion and non-early cardioversion under the frequentist framework.

*The antiarrhythmic drugs as early pharmacologic cardioversion in the study included amiodarone, propafenone, flecainide, vernakalant, vanoxerine, and sotalol.

†All endpoints are efficacy endpoints, including cardioversion to sinus rhythm within 24 hours, maintenance in sinus rhythm within 1-week and maintenance in sinus rhythm within 1-4 weeks, safety endpoints including bradycardia, tachyarrhythmia, hypotension, gastrointestinal disorders, and nervous system disorders, and prognostic endpoints including all-cause mortality, stroke or TIA and heart failure.

Abbreviations: CI: confidence Intervals; TIA: Transient Ischemic Attack; Amio: Amiodarone; Sota: Sotalol; Vern: Vernakalant; Vano: Vanoxerine; Prop: Propafenone; Flec: Flecainide; NA: not available.

Supplemental Figure 2. Pair-wise meta-analysis of all endpoints

*The antiarrhythmic drugs as early pharmacologic cardioversion in the study included amiodarone, propafenone, flecainide, vernakalant, vanoxerine, and sotalol.

†All endpoints are efficacy endpoints including cardioversion to sinus rhythm within 24 hours, maintenance in sinus rhythm within 1-week and maintenance in sinus rhythm within 1-4 weeks, safety endpoints including bradycardia, tachyarrhythmia, hypotension, gastrointestinal disorders, and nervous system disorders, and prognostic endpoints including all-cause mortality, stroke or TIA and heart failure.

Abbreviations: CI: confidence Intervals; TIA: Transient Ischemic Attack.

Supplemental Figure 3. The funnel plots of standard errors vs. effect estimates for publication bias and study effects. Data are presented only when the number of studies for an endpoint was >=10.

*The endpoints with the number of studies >=10 are efficacy endpoints including cardioversion to sinus rhythm within 24 hours, safety endpoints including bradycardia, hypotension, and gastrointestinal disorders.

Supplemental Figure 4. Subgroup analyses in recent-onset atrial fibrillation patients according to continuing cardioversion for more than 24 hours after immediate cardioversion and cardioversion deliveries.
Abbreviations: CrI: Credible Interval.

Supplemental Table 1. Definition of clinical endpoints of the included RCTs.
Abbreviations: RCT: Randomized Controlled Trials; TIA: Transient Ischemic Attack; ECG: Electrocardiograph; SR: Sinus Rhythm; AF: Atrial Fibrillation; MAP: Mean Arterial Pressure; VT: Ventricular Tachycardia; bpm: beat per minute; NA: not available.

Supplemental Table 2. GRADE quality of evidence of all endpoints using CINeMA.
[1]Within-study bias (ie, risk of bias in the included studies),[2]Across-study bias (ie, publication and reporting bias), [3]Indirectness, [4]Imprecision: confidence intervals include values that lead into different clinical Decisions; [5]Heterogeneity,[6]Incoherence (ie, differences between direct and indirect evidence)
Abbreviations: TIA: Transient Ischemic Attack; Amio: Amiodarone; Sota: Sotalol; Vern: Vernakalant; Vano: Vanoxerine; Prop: Propafenone; Flec: Flecainide; NA: not available.

Supplemental Table 3. Assessment of heterogeneity and consistency, for the safety endpoints among different antiarrhythmic drugs as early pharmacologic cardioversion.
Abbreviations: TIA: Transient Ischemic Attack; Amio: Amiodarone; Sota: Sotalol; Vern: Vernakalant; Vano: Vanoxerine; Prop: Propafenone; Flec: Flecainide; NA: not available.

Supplemental Table 4. Assessment of Bayesian random-effects model fit and inconsistency, for all endpoints in the study group with early pharmacologic cardioversion arm as different antiarrhythmic drugs.
*Once total residual variance approximated the number of data points, it means a good model fit; †DIC refers to deviance information criterion, lower values of DIC are better.
Abbreviations: TIA: Transient Ischemic Attack; Amio: Amiodarone; Sota: Sotalol; Vern: Vernakalant; Vano: Vanoxerine; Prop: Propafenone; Flec: Flecainide; DIC: Deviance Information criterion; NA: not available.

Systematic Review Searching Record

Literature search details

Language Restriction: none

**Databases and Trial registers:**	**Citations**
Databases:	
Medline	414
Embase	1805
Pubmed	793
Cochrane Library	1111
Web of Science	4100
Total (databases)	8223
After de-duplication:	4932
Trial registers:	
USA(ClinicalTrials.gov)	237
EU/EEA(Clinicaltrialsregister.eu)	52
Total (trial registers)	289

Individual strategies

OVID-MEDLINE

#	Searches	Results
1	Atrial Fibrillation/	88241
2	(Fibrillation* and A$trial).ab,ti.	88247
3	(Auricular Fibrillation*).ab,ti.	2300
4	(Familial Atrial Fibrillation*).ab,ti.	430
5	1 or 2 or 3 or 4	88414
6	Electric Countershock/	15114
7	(Electric Countershock*).ab,ti.	15117
8	(Cardioversion).ab,ti.	6332
9	(Cardioversions).ab,ti	317
10	(Electric Defibrillation*).ab,ti	4358
11	(Electrical and Cardioversion*).ab,ti	2144
12	(Electroversion and Cardiac).ab,ti	18
13	(Electroversion and Therapy).ab,ti	27
14	6 or 7 or 8 or 9 or 10 or 11 or 12 or 13	18364
15	5 and 14	6562
16	letter/	102941
17	editorial/	66374
18	news/	25170
19	comment/	99398
20	case report/	1284483
21	16 or 17 or 18 or 19 or 20	1559271
22	randomized controlled trial/ or random$.ti,ab.	785019
23	21 not 22	1529380
24	(rat or rats or mouse or mice).ti.	1356341
25	23 or 24	2881004
26	15 not 25	6241
27	randomized controlled trial	422084
28	26 and 27	414

Embase

#	Searches	Results
1	'atrial fibrillation'/exp	181533
2	'auricular fibrilation':ab,ti	11
3	'auricular fibrillation':ab,ti	954
4	'cardiac atrial fibrillation':ab,ti	14
5	'cardiac atrium fibrillation':ab,ti	1
6	'heart atrial fibrillation':ab,ti	15
7	'heart atrium fibrillation':ab,ti	17
8	'non-valvular atrial fibrillation':ab,ti	4450
9	'nonvalvular atrial fibrillation':ab,ti	3082
10	#1 OR #2 OR #3 OR #4 OR #5 OR #6 OR #7 OR #8 OR #9	182144
11	'cardioconversion'/exp	22890
12	'cardioconversion':ab,ti	12
13	'cardioconversion':ab,ti AND 'electric':ab,ti	0
14	'counter shock':ab,ti	52
15	'countershock':ab,ti	508
16	'conversion':ab,ti AND 'electric':ab,ti	1509
17	'electric countershock':ab,ti	78
18	'electrical cardioversion':ab,ti	3045
19	'electrocardioversion':ab,ti	103
20	'electroconversion':ab,ti	85
21	#11 OR #12 OR #13 OR #14 OR #15 OR #16 OR #17 OR #18 OR #19 OR #20	24882
22	#10 AND #21	12354
23	'letter'/exp	1132251
24	'editorial'/exp	686094
25	'literature'/exp	254534
26	'case report'/exp	2763124
27	letter:ti OR comment*:ti	224961
28	#23 OR #24 OR #25 OR #26 OR #27	4662485
29	'randomized controlled trial' OR 'randomized controlled trials as topic'/exp OR random*:ti,ab	1931942
30	#28 NOT #29	4597356
31	'animals'/exp NOT 'humans'/exp	5694704
32	'animals, laboratory'/exp	747833
33	'animal experiment'/exp	277590

34	'animal model'/exp	1542615
35	'rodent'/exp	4010258
36	rat:ti OR rats:ti OR mouse:ti OR mice:ti	1659798
37	#30 OR #31 OR #32 OR #33 OR #34 OR #35 OR #36	11360287
38	#22 NOT #37	9660
39	'randomized controlled trial':pt OR 'randomized controlled trial'/exp OR 'randomized controlled trials as topic'/exp	897867
40	'controlled clinical trial'/de	435546
41	randomized:ab	782626
42	placebo:ab	323714
43	randomly:ab	490089
44	'clinical trials as topic'/de	114216
45	trial:ti	349731
46	#40 OR #41 OR #42 OR #43 OR #44 OR #45 OR #46	1986230
47	#38 AND #46	1805

Pubmed

#	Searches	Results
1	Search Atrial Fibrillation[mh]	62488
2	Search Atrial Fibrillation[Title/Abstract]	80397
3	Search Atrial Fibrillations[Title/Abstract]	119
4	Search ((Atrial[Title/Abstract] AND Fibrillation*[Title/Abstract]))	81689
5	Search ((Atrial[Title/Abstract] AND Fibrillations*[Title/Abstract]))	160
6	Search Auricular Fibrillation[Title/Abstract]	1549
7	Search Auricular Fibrillations[Title/Abstract]	7
8	Search ((Auricular[Title/Abstract] AND Fibrillation*[Title/Abstract]))	1872
9	Search ((Auricular[Title/Abstract] AND Fibrillations*[Title/Abstract]))	11
10	Search Familial Atrial Fibrillation[Title/Abstract]	114
11	Search Familial Atrial Fibrillations[Title/Abstract]	7
12	Search (1 or 2 or 3 or 4 or 5 or 6 or 7 or 8 or 9 or 10 or 11)	93872
13	Search Cardioversion[mh]	15426
14	Search Electric Countershock[Title/Abstract]	401
15	Search Electric Countershocks[Title/Abstract]	5
16	Search ((Electric[Title/Abstract] AND Countershock[Title/Abstract]))	416
17	Search ((Electric[Title/Abstract] AND Countershocks[Title/Abstract]))	9
18	Search Electric Defibrillation[Title/Abstract]	2
19	Search Electric Defibrillations[Title/Abstract]	96
20	Search ((Electric[Title/Abstract] AND Defibrillation[Title/Abstract]))	432
21	Search ((Electric[Title/Abstract] AND Defibrillations[Title/Abstract]))	16
22	Search Electrical Cardioversion[Title/Abstract]	1748
23	Search Electrical Cardioversions[Title/Abstract]	59
24	Search ((Electrical[Title/Abstract] AND Cardioversion[Title/Abstract]))	2196
25	Search ((Electrical[Title/Abstract] AND Cardioversions[Title/Abstract]))	115
26	Search Cardiac Electroversion[Title/Abstract]	2

27	Search ((Cardiac[Title/Abstract] AND Electroversion[Title/Abstract]))	11
28	Search Electroversion Therapy[Title/Abstract]	5
29	Search Electroversion Therapies[Title/Abstract]	1
30	Search (13 or 14 or 15 or 16 or 17 or 18 or 19 or 20 or 21 or 22 or 23 or 24 or 25 or 26 or 27 or 28 or 29)	16508
31	Search (12 and 30)	5237
32	Search (animals[mh]) NOT humans[mh]	4913840
33	Search Animals, Laboratory[mh]	920957
34	Search Animal Experimentation[mh]	9914
35	Search Models, Animal[mh]	610104
36	Search rodentia[mh]	3379623
37	Search (rat[Title] OR rats[Title] OR mouse[Title] OR mice[Title])	1381045
38	Search (32 or 33 or 34 or 35 or 36 or 37)	5898621
39	Search (31 not 38)	5136
40	Search ((randomized controlled trial[Publication Type]) OR randomized controlled trial[mh]) OR Randomized Controlled Trials as Topic[mh]	697218
41	Search controlled clinical trial[Publication Type]	641096
42	Search (randomized[Title/Abstract]) OR randomised[Title/Abstract]	699428
43	Search placebo[Title/Abstract]	229322
44	Search randomly[Title/Abstract]	370337
45	Search trial[Title]	250705
46	Search (40 or 41 or 42 or 43 or 44 or 45)	1374088
47	Search (39 and 46)	793

Cochrane Library [Wiley]

ID	Searches	Results
#1	[mh "Atrial Fibrillation"]	4943
#2	(Atrial Fibrillation*):ti,ab,kw	13782
#3	(Auricular Fibrillation*):ti,ab,kw	71
#4	{or #1-#3}	13786
#5	[mh "Cardioversion"]	894
#6	(Cardioversion*):ti,ab,kw	1621
#7	(Countershock*):ti,ab,kw and (Electric):ti,ab,kw	900
#8	(Defibrillation*):ti,ab,kw and (Electric):ti,ab,kw	311
#9	(Cardioversion*):ti,ab,kw and (Electric):ti,ab,kw	475
#10	(Electroversion*):ti,ab,kw and (Cardiac):ti,ab,kw	1
#11	(Electroversion):ti,ab,kw and (Therapy):ti,ab,kw	1
#12	{or #5-#11}	2140
#13	#4 and #12	1426
#14	[mh animals] not [mh humans]	59
#15	rat or rats or mouse or mice:ti,ab,kw	14608
#16	#14 or #15	14663
#17	#13 not #16	1422
#18	randomized:ti,ab,kw	982674
#19	placebo:ti,ab,kw	327077
#20	randomly:ti,ab,kw	271144
#21	clinical trials:ti,ab,kw	189408
#22	{or #18-#21}	1206445
#23	#17 and #22	1111

Web of Science [Thompson Reuters]

#	Searches	Results
1	TS=Atrial Fibrillation	141868
2	TS=（Atrial Fibrillation*）	141876
3	TS=（Fibrillation* and A$trial）	141888
4	TS=（Auricular Fibrillation*）	3151
5	TS=（Familial Atrial Fibrillation*）	839
6	#5 OR #4 OR #3 OR #2 OR #1	142175
7	TS=Cardioversion	10587
8	TS=（Electric and Countershock*）	15227
9	TS=（Electric and Defibrillation*）	5379
10	TS=（Electrical and Cardioversion*）	3909
11	TS=（Electroversion and Cardiac）	19
12	TS=Cardioversions	363
13	TS=（Electroversion and Therapy）	29
14	#13 OR #12 OR #11 OR #10 OR #9 OR #8 OR #7	22625
15	#14 AND #6	10068
16	TS= clinical trial* OR TS=research design OR TS=comparative stud* OR TS=evaluation stud* OR TS=controlled trial* OR TS=follow-up stud* OR	10472334
17	#16 AND #15	4100

Clinical Trials.gov

Basic search

237 Studies found for: (Atrial Fibrillation OR Auricular Fibrillation) AND (Cardioversion OR Electric Countershock OR Electric Defibrillation OR Electrical Cardioversion OR Electroversion Therapy)

Clinical trials register. eu

Basic search

52 Studies found for: Cardioversion AND Atrial Fibrillation

Supplemental Figure 1. Network meta-analysis results for all endpoints between early pharmacologic cardioversion and non-early cardioversion under the frequentist framework.

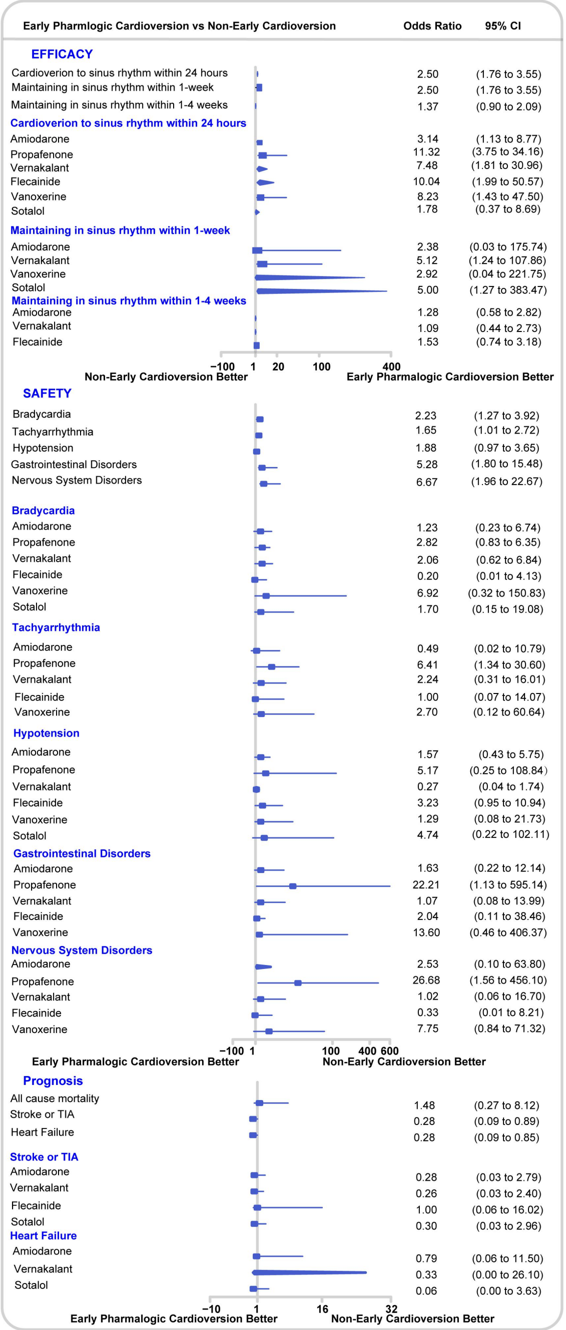

Supplemental Figure 1

*The antiarrhythmic drugs as early pharmacologic cardioversion in the study included amiodarone, propafenone, flecainide, vernakalant, vanoxerine, and sotalol.

†All endpoints are efficacy endpoints, including cardioversion to sinus rhythm within 24 hours, maintenance in sinus rhythm within 1-week and maintenance in sinus rhythm within 1-4 weeks, safety endpoints including bradycardia, tachyarrhythmia, hypotension, gastrointestinal disorders, and nervous system disorders, and prognostic endpoints including all-cause mortality, stroke or TIA and heart failure.

Abbreviations: CI: confidence Intervals; TIA: Transient Ischemic Attack.

Supplemental Figure 2. Pair-wise meta-analysis of all endpoints

Supplemental Figure 2

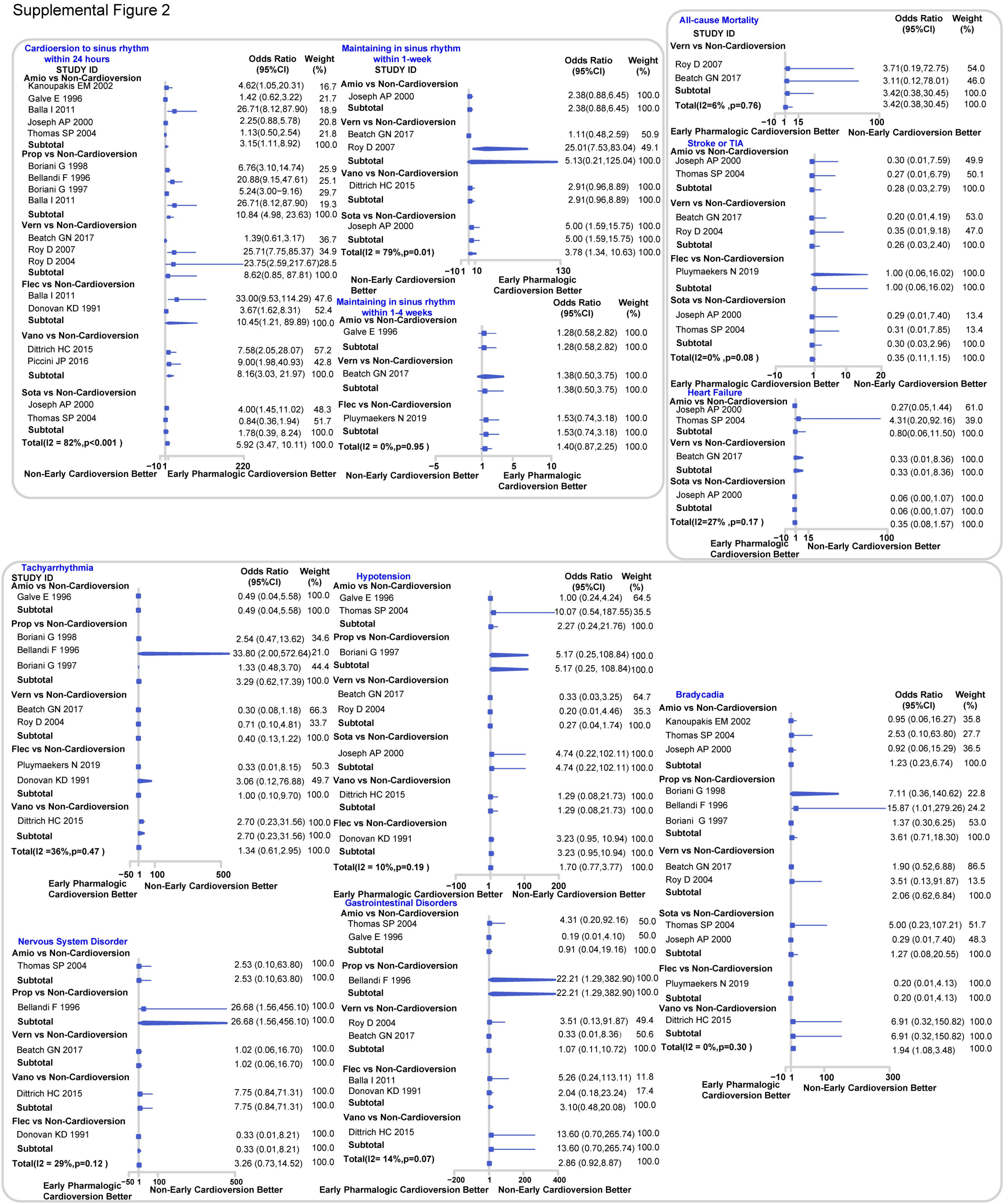

*The antiarrhythmic drugs as early pharmacologic cardioversion in the study included amiodarone, propafenone, flecainide, vernakalant, vanoxerine, and sotalol.

†All endpoints are efficacy endpoints including cardioversion to sinus rhythm within 24 hours, maintenance in sinus rhythm within 1-week and maintenance in sinus rhythm within 1-4 weeks, safety endpoints including bradycardia, tachyarrhythmia, hypotension, gastrointestinal disorders, and nervous system disorders, and prognostic endpoints including all-cause mortality, stroke or TIA and heart failure.

Abbreviations: CI: confidence Intervals; TIA: Transient Ischemic Attack; Amio: Amiodarone; Sota: Sotalol; Vern: Vernakalant; Vano: Vanoxerine; Prop: Propafenone; Flec: Flecainide; NA: not available.

Supplemental Figure 3. The funnel plots of standard errors vs. effect estimates for publication bias and study effects. Data are presented only when the number of studies for an endpoint was >=10.

Supplemental Figure 3

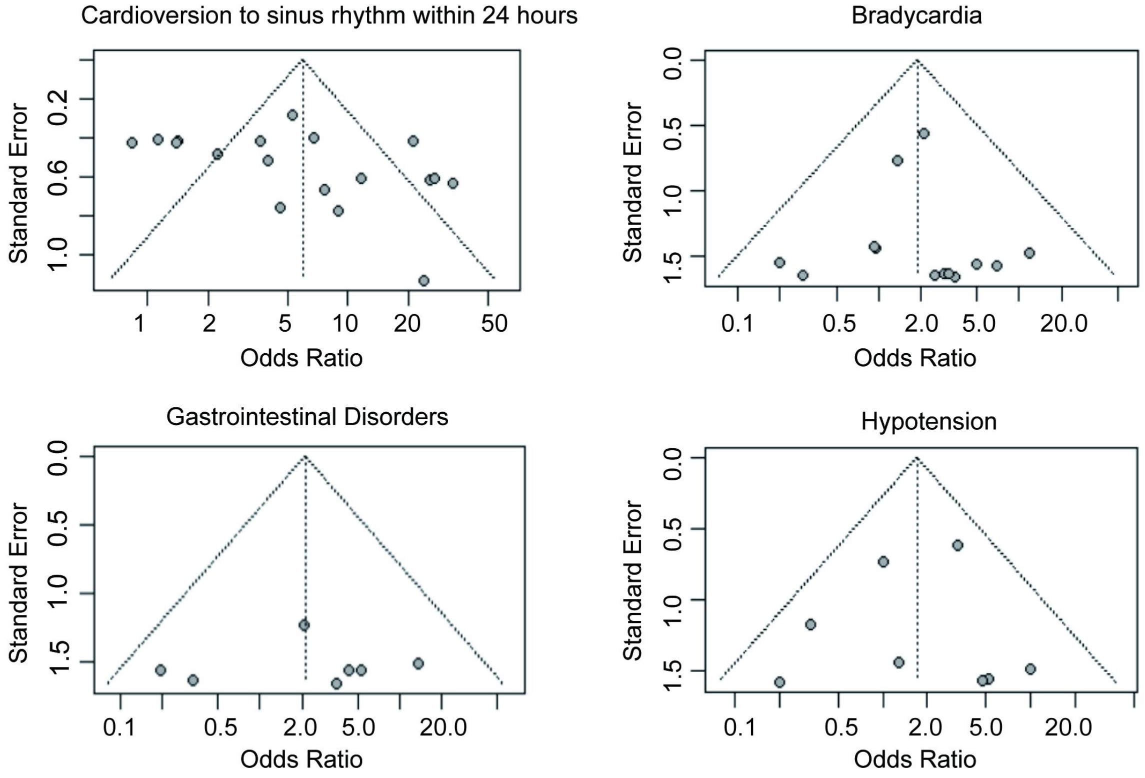

[*]The endpoints with the number of studies >=10 are efficacy endpoints including cardioversion to sinus rhythm within 24 hours, safety endpoints including bradycardia, hypotension, and gastrointestinal disorders.

Supplemental Figure 4. Subgroup analyses in recent-onset atrial fibrillation patients according to continuing cardioversion for more than 24 hours after immediate cardioversion and cardioversion deliveries.

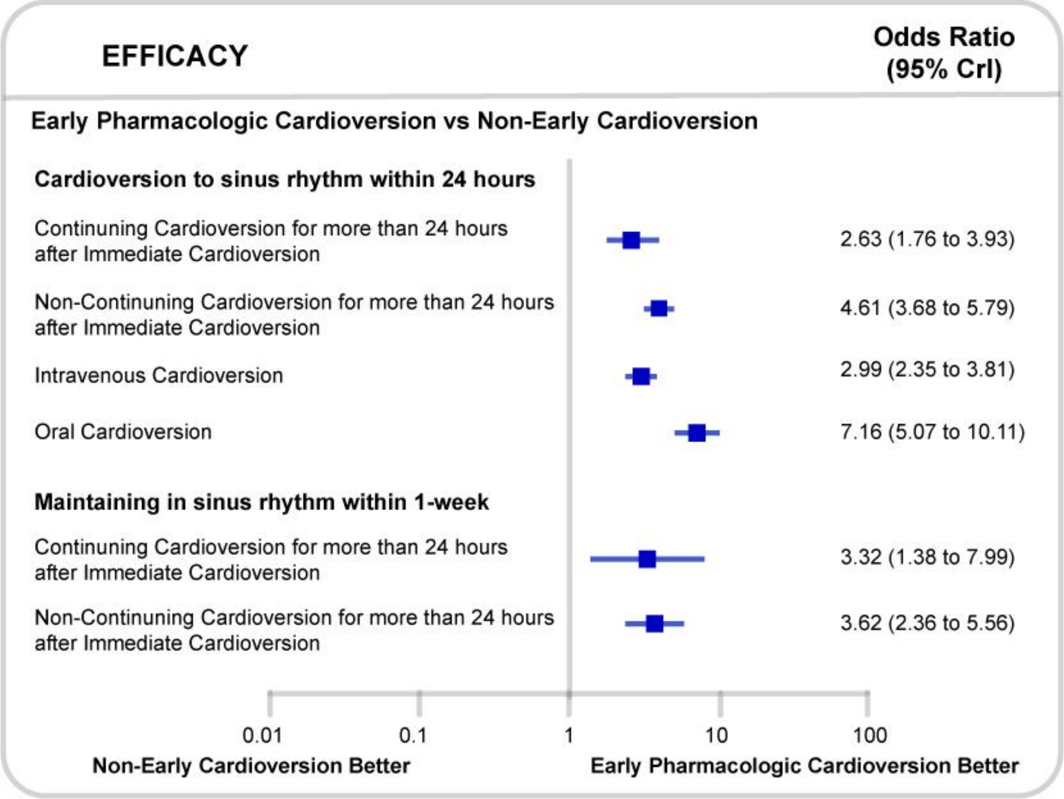

Supplemental Figure 4

Abbreviations: CrI: Credible Interval.

Supplemental Table 1. Definition of clinical endpoints of the included RCTs.

Trial	Cardioversion to sinus rhythm within 24 hours	Maintaining in sinus rhythm within 1-week	Maintaining in sinus rhythm within 1-4 weeks	Bradycardia	Tachyarrhythmia
Kanoupakis EM 2003	Conversion to normal sinus rhythm within the 24 hours after infusion according to the standard method(Clinical Cardiac Electrophysiology vs Techniques and Interpretations, 2nd Philadelphia)	NA	NA	ECGs were recorded at a paper speed of 50 mm/s. The longest QT interval in any of the leads was measured and averaged from three consecutive QRS-T complexes. The QT interval was corrected (QTc) for rate using Bazett's formula	NA
Boriani G 1998	Conversion ta sinus rhythm within 3 hours and within 8 hours in patients via electrocardiogram recorded every hour for 4 hours and then every 2 hours for the next 4 hours.	NA	NA	Bradycardia and pauses > 2 seconds at Holter	Sustained atrial flutter or atrial tachycardia at Holter
Donovan KD 1991	Stable sinus rhythm until the end of the 6-hour monitoring period.	NA	NA	NA	NA
Hohnloser SH 2004	No continuous ECG monitoring 24 hours after drug infusion	NA	NA	Bradycardia and first-degree atrioventricular block	The torsade de Pointes monitoring via continuous ECG.
Roy D 2004	Twelve-lead ECGs were obtained before dosing and every minute during infusion to 5 min after, as well as at 24-h	NA	NA	Heart rate was less than 50 beats/min, a new atrioventricular or bundle-branch block via continuous ECG.	Any polymorphic ventricular tachycardia via continuous ECG.
Dittrich HC 2015	Restoration of SR (at least 1 continuous minute)through 4 hours or 24 hours after the study drug was determined from the 12-lead ECGs, telemetry, and Holter recordings	On day 7, the proportion of subjects in the placebo group in SR	NA	Sinus pause >3 seconds and sinus bradycardia via 12-lead ECG and Holter throughout the monitoring period	Nonsustained monomorphic ventricular tachycardia (VT) (6–29 beats), sustained monomorphic VT (30þ beats), polymorphic VT (any duration) were observed on Holter throughout the monitoring period
Thomas SP 2004	Patients remaining in atrial fibrillation after 12 hours were referred for electrical cardioversion.	NA	NA	NA	NA
Galve E 1996	Revert to sinus rhythm.	NA	Atrial fibrillation recurred during a 15-day follow-up	NA	Nonsustained ventricular tachycardia
Roy D 2007	Conversion to sinus rhythm for at least 1 minute. The absence of AF or atrial flutter, which included conversion to sinus rhythm and a paced rhythm. All 12-lead ECGs and 24-hour Holter recordings were reviewed by a cardiologist at the central ECG laboratory.	Converted to sinus rhythm relapsed to AF at 24 hours	NA	NA	NA

Bellandi F 1996	conversion to SR within 24 hours via the analysis of QRS and QTc (derived from Bazett's formula)was expressed as the mean of three consecutive cardiac cycles of the ECG strips.	NA	NA	NA	All 12-lead ECGs and 24-hour Holter recordings were reviewed. Ventricular tachycardia was defined as 3 wide-complex beats with a rate of 100 bpm.
Boriani G 1997	A 24-hour Holter monitor was applied; after 1 to 2 hours of observation to assess the stability of atrial fibrillation.	NA	NA	NA	NA
Balla I 2011	12-lead electrocardiograms were recorded before drug ingestion and at 3, 6, 12, and 24 hours thereafter.	NA	NA	NA	NA
Piccini JP 2016	Conversion to sinus rhythm through 24 hours on ECG	NA	NA	NA	NA
Joseph AP 2000	All patients had ECG monitoring throughout the 24-hour trial period, and the time of rhythm reversion and ventricular rate on reversion to sinus rhythm as was noted.	All patients had ECG monitoring throughout the 48-hour trial period, time of rhythm reversion and ventricular rate on reversion to sinus rhythm were noted.	NA	Ventricular rate <40 beats/min either in AF or sinus rhythm, or ventricular rate <60 beats/min with MAP <70 mm Hg	Torsade de Pointes polymorphic ventricular tachycardia or other.
Pluymaekers N 2019	NA	NA	The presence of sinus rhythm on ECG was recorded at the 4-week trial visit. All ECGs were centrally assessed for the presence of sinus rhythm by the first two authors	Leading to an emergency department visit or hospital admission due to bradycardia.	Leading to an emergency department visit or hospital admission due to tachyarrhythmia.
Beatch GN 2017	Conversion from atrial fibrillation to sinus rhythm in the 24 hours after the start of infusion of placebo or vernakalant via 12-lead ECG.	The proportions of patients who were in SR at 7 days after first exposure	Reporting no AF symptoms in the 30 days after the first infusion	Bradycardia, sinus bradycardia, and sinus arrest were captured identified from Holter monitoring data collected.	Events of clinical interest, regardless of severity.

Abbreviations: RCT: Randomized Controlled Trials; TIA: Transient Ischemic Attack; ECG: Electrocardiograph; SR: Sinus Rhythm; AF: Atrial Fibrillation; MAP: Mean Arterial Pressure; VT: Ventricular Tachycardia; bpm: beat per minute; NA: not available.

Supplemental Table 1. Definition of clinical endpoints of the included RCTs (continued).

Trial	Hypotension	Gastrointestinal Disorders	Nervous System Disorders	All-caused Mortality	Stroke or TIA	Heart Failure
Kanoupakis EM 2003	NA	NA	NA	NA	NA	NA
Boriani G 1998	NA	NA	NA	NA	NA	NA
Donovan KD 1991	A decrease in systolic arterial pressure of one-third or more of the initial measurement.	Nausea	Hot flushes	NA	NA	NA
Hohnloser SH 2004	NA	NA	NA	One patient died before the start of the infusion from a recurrent pulmonary embolism caused by postoperative immobility. The other patient died from pancreatic carcinoma leading to respiratory failure 18 days after the start of infusion.	NA	NA
Roy D 2004	Persistent and transient hypotension	Nausea	NA	NA	A transient cerebral ischemic attack occurred one day after conversion	NA
Dittrich HC 2015	Vascular disorder hypotension	Nausea	Dizziness; Headache.	NA	NA	NA
Thomas SP 2004	Symptomatic hypotension(Blood pressure<90mmHg)	Nausea and vomiting.	Paraesthesia	NA	An embolic stroke	NA
Galve E 1996	Hypotension below 100 mm Hg	Vomit	NA	NA	NA	NA
Roy D 2007	NA	NA	NA	One patient with lung cancer died of pneumonia.	NA	NA
Bellandi F 1996	NA	Digestive side effects (especially nausea)	dizziness, and headache	NA	NA	NA
Boriani G 1997	Hypotension	NA	NA	NA	NA	NA
Balla I 2011	NA	Diarrhea.	NA	NA	NA	NA
Piccini JP 2016	NA	NA	NA	NA	NA	NA
Joseph AP 2000	Mean arterial pressure <70mmHg.	NA	NA	NA	Stroke	New inspiratory crepitations with signs of pulmonary venous congestion on chest radiograph.

Pluymaekers N 2019		NA	NA	NA	Leading to an emergency department visit or hospital admission due to ischemic stroke, transient ischemic attack.	Leading to an emergency department visit or hospital admission due to heart failure.
Beatch GN 2017	Systolic blood pressure<85mmHg; Clinically significant hypotension as assessed by the investigator.	Diarrhea	Dizziness; Dysgeusia.	One patient with a history of abdominal aortic aneurysm, heart failure, idiopathic pulmonary fibrosis, rectal cancer, and pulmonary tuberculosis died on day 6.	Cerebral infarction	Congestive heart failure

Abbreviations: RCT: Randomized Controlled Trials; TIA: Transient Ischemic Attack; ECG: Electrocardiograph; SR: Sinus Rhythm; AF: Atrial Fibrillation; MAP: Mean Arterial Pressure; VT: Ventricular Tachycardia; bpm: beat per minute; NA: not available.

Table S2. GRADE quality of evidence of all endpoints using CINeMA.

Endpoints	Comparisons	Direct evidence Quality of evidence	Indirect evidence Quality of evidence	Network meta-analysis Quality of evidence
	**Amio vs Flec**	⊕ ⊕ ⊕ ⊕ High	⊕ ⊕ ⊕⊖ Moderate[3,5]	⊕ ⊕ ⊕⊖ Moderate [5]
	**Amio vs Prop**	⊕ ⊕ ⊕ ⊕ High	⊕ ⊕ ⊕⊖ Moderate[3,5]	⊕ ⊕ ⊕⊖ Moderate [5]
	**Amio vs Sota**	⊕ ⊕ ⊕ ⊕ High	⊕ ⊕ ⊕⊖ Moderate[3,5]	⊕ ⊕ ⊕⊖ Moderate [5]
	**Flec vs Non-Early Cardioversion**	⊕ ⊕ ⊕ ⊕ High	⊕ ⊕ ⊕⊖ Moderate[3,5]	⊕ ⊕ ⊕⊖ Moderate [5]
	**Flec vs Prop**	⊕ ⊕ ⊕ ⊕ High	⊕ ⊕ ⊕⊖ Moderate[3,5]	⊕ ⊕ ⊕⊖ Moderate [5]
	**Non-Early Cardioversion vs Prop**	⊕ ⊕ ⊕ ⊕ High	⊕ ⊕ ⊕⊖ Moderate[3,5]	⊕ ⊕ ⊕⊖ Moderate [5]
	**Non-Early Cardioversion vs Sota**	⊕ ⊕ ⊕ ⊕ High	⊕ ⊕ ⊕⊖ Moderate[3,5]	⊕ ⊕ ⊕⊖ Moderate [5]
	**Non-Early Cardioversion vs Vano**	⊕ ⊕ ⊕⊖ Moderate[5]	NA	NA
	**Non-Early Cardioversion vs Vern**	⊕ ⊕ ⊕ ⊕ High	NA	NA
**Cardioversion to sinus rhythm within 24 hours**	**Amio vs Vano**	NA	⊕ ⊕⊖ ⊖ Low[2,3,4]	NA
	**Amio vs Vern**	NA	⊕ ⊕⊖ ⊖ Low[2,3,4]	NA
	**Flec vs Sota**	NA	⊕ ⊕⊖ ⊖ Low[2,3,4]	NA
	**Flec vs Vano**	NA	⊕ ⊕⊖ ⊖ Low[2,3,4]	NA
	**Flec vs Vern**	NA	⊕ ⊕⊖ ⊖ Low[2,3,4]	NA
	**Prop vs Sota**	NA	⊕ ⊕⊖ ⊖ Low[2,3,4]	NA
	**Prop vs Vano**	NA	⊕ ⊕⊖ ⊖ Low[2,3,4]	NA
	**Prop vs Vern**	NA	⊕ ⊕⊖ ⊖ Low[2,3,4]	NA
	**Sota vs Vano**	NA	⊕ ⊕⊖ ⊖ Low[2,3,4]	NA
	**Sota vs Vern**	NA	⊕ ⊕⊖ ⊖ Low[2,3,4]	NA
	**Vano vs Vern**	NA	⊕ ⊕⊖ ⊖ Low[2,3,4]	NA

	**Sota vs Non-Early Cardioversion**	⊕⊕⊕⊝ Moderate[2]	⊕⊕⊕⊝ Moderate[3]	⊕⊕⊕⊝ Moderate[3]
**Maintaining in sinus rhythm within 1-week**	**Amio vs Non-Early Cardioversion**	⊕⊕⊕⊝ Moderate[2]	⊕⊕⊕⊝ Moderate[3]	⊕⊕⊕⊝ Moderate[3]
	**Sota vs Amio**	⊕⊕⊕⊝ Moderate[2]	⊕⊕⊕⊝ Moderate[3]	⊕⊕⊕⊝ Moderate[3]
	**Amio vs Vano**	NA	⊕⊕⊝⊝ Low[3,4,5]	NA
	**Amio vs Vern**	NA	⊕⊕⊝⊝ Low[3,4,5]	NA
	**Sota vs Vano**	NA	⊕⊕⊝⊝ Low[3,4,5]	NA
	**Sota vs Vern**	NA	⊕⊕⊝⊝ Low[3,4,5]	NA
	**Vano vs Vern**	NA	⊕⊕⊝⊝ Low[3,4,5]	NA
	**Non-Early Cardioversion vs Vano**	⊕⊕⊝⊝ Low[1,5]	NA	NA
	**Non-Early Cardioversion vs Vern**	⊕⊕⊝⊝ Low[1,5]	NA	NA
**Maintaining in sinus rhythm within 1-4 weeks**	**Flec vs Non-Early Cardioversion**	⊕⊕⊕⊝ Moderate[5]	NA	NA
	**Amio vs Non-Early Cardioversion**	⊕⊕⊝⊝ Low[1,5]	NA	NA
	**Vern vs Non-Early Cardioversion**	⊕⊕⊝⊝ Low[1,5]	NA	NA
	**Amio vs Flec**	NA	⊕⊕⊝⊝ Low[3,4,5]	NA
	**Amio vs Vern**	NA	⊕⊕⊝⊝ Low[3,4,5]	NA
	**Flec vs Vern**	NA	⊕⊕⊝⊝ Low[3,4,5]	NA
	**Amio vs Non-Early Cardioversion**	⊕⊕⊝⊝ Low[1,5]	⊕⊕⊝⊝ Low[3,4,5]	⊕⊕⊝⊝ Low[4,5,6]
	**Amio vs Sota**	⊕⊕⊝⊝ Low[1,5]	⊕⊕⊝⊝ Low[3,4,5]	⊕⊕⊝⊝ Low[4,5,6]
	**Non-Early Cardioversion vs Sota**	⊕⊕⊝⊝ Low[1,5]	⊕⊕⊝⊝ Low[3,4,5]	⊕⊕⊝⊝ Low[4,5,6]
	**Non-Early Cardioversion vs Vano**	⊕⊕⊝⊝ Low[1,5]	NA	NA
	**Non-Early Cardioversion vs Vern**	⊕⊕⊝⊝ Low[1,5]	NA	NA

[1]Within-study bias (ie, risk of bias in the included studies),[2]Across-study bias (ie, publication and reporting bias), [3]Indirectness, [4]Imprecision: confidence intervals include values that lead into different clinical

Decisions; [5]Heterogeneity,[6]Incoherence (ie, differences between direct and indirect evidence)

Abbreviations: TIA: Transient Ischemic Attack; Amio: Amiodarone; Sota: Sotalol; Vern: Vernakalant; Vano: Vanoxerine; Prop: Propafenone; Flec: Flecainide; NA: not available.

Table S2. GRADE quality of evidence of all endpoints using CINeMA (continued).

Endpoints	Comparisons	Direct evidence<br>Quality of evidence	Indirect evidence<br>Quality of evidence	Network meta-analysis<br>Quality of evidence
Bradycardia	Amio vs Flec	NA	⊕ ⊕⊝ ⊝ Low[4,5]	NA
	Amio vs Prop	NA	⊕ ⊕⊝ ⊝ Low[4,5]	NA
	Amio vs Vano	NA	⊕ ⊕⊝ ⊝ Low[4,5]	NA
	Amio vs Vern	NA	⊕ ⊕⊝ ⊝ Low[4,5]	NA
	Flec vs Prop	NA	⊕ ⊕⊝ ⊝ Low[4,5]	NA
	Flec vs Sota	NA	⊕ ⊕⊝ ⊝ Low[4,5]	NA
	Flec vs Vano	NA	⊕ ⊕⊝ ⊝ Low[4,5]	NA
	Flec vs Vern	NA	⊕ ⊕⊝ ⊝ Low[4,5]	NA
	Prop vs Sota	NA	⊕ ⊕⊝ ⊝ Low[4,5]	NA
	Prop vs Vern	NA	⊕ ⊕⊝ ⊝ Low[4,5]	NA
	Sota vs Vano	NA	⊕ ⊕⊝ ⊝ Low[4,5]	NA
	Sota vs Vern	NA	⊕ ⊕⊝ ⊝ Low[4,5]	NA
	Vano vs Vern	NA	⊕ ⊕⊝ ⊝ Low[4,5]	NA
	Flec vs Non-Early Cardioversion	⊕ ⊕ ⊕⊝ Moderate[5]	NA	NA
	Non-Early Cardioversion vs Prop	⊕ ⊕ ⊕⊝ Moderate[5]	NA	NA
Tachycardia	Amio vs Non-Early Cardioversion	⊕ ⊕ ⊕⊝ Moderate[5]	NA	NA
	Flec vs Non-Early Cardioversion	⊕ ⊕ ⊕⊝ Moderate[5]	NA	NA
	Non-Early Cardioversion vs Prop	⊕ ⊕ ⊕⊝ Moderate[5]	NA	NA
	Non-Early Cardioversion vs Vano	⊕ ⊕ ⊕⊝ Moderate[5]	NA	NA
	Non-Early Cardioversion vs Vern	⊕ ⊕ ⊕⊝ Moderate[5]	NA	NA
	Amio vs Flec	NA	⊕ ⊕⊝ ⊝ Low[4,5]	NA

	**Amio vs Prop**	NA	⊕ ⊕⊝ ⊝ Low[4,5]	NA
	**Amio vs Vano**	NA	⊕ ⊕⊝ ⊝ Low[4,5]	NA
	**Amio vs Vern**	NA	⊕ ⊕⊝ ⊝ Low[4,5]	NA
	**Flec vs Prop**	NA	⊕ ⊕⊝ ⊝ Low[4,5]	NA
	**Flec vs Vano**	NA	⊕ ⊕⊝ ⊝ Low[4,5]	NA
	**Flec vs Vern**	NA	⊕ ⊕⊝ ⊝ Low[4,5]	NA
	**Prop vs Vano**	NA	⊕ ⊕⊝ ⊝ Low[4,5]	NA
	**Prop vs Vern**	NA	⊕ ⊕⊝ ⊝ Low[4,5]	NA
	**Vano vs Vern**	NA	⊕ ⊕⊝ ⊝ Low[4,5]	NA
**Hypotension**	**Amio vs Non-Early Cardioversion**	⊕ ⊕ ⊕⊝ Moderate[5]	⊕ ⊕ ⊕⊝ Moderate[3,5]	⊕ ⊕⊝ ⊝ Low[3,5,6]
	**Amio vs Sota**	⊕ ⊕ ⊕⊝ Moderate[5]	⊕ ⊕ ⊕⊝ Moderate[3,5]	⊕ ⊕⊝ ⊝ Low[3,5,6]
	**Non-Early Cardioversion vs Sota**	⊕ ⊕ ⊕⊝ Moderate[5]	⊕ ⊕ ⊕⊝ Moderate[3,5]	⊕ ⊕⊝ ⊝ Low[3,5,6]
	**Non-Early Cardioversion vs Prop**	⊕ ⊕ ⊕⊝ Moderate[5]	NA	NA
	**Flec vs Non-Early Cardioversion**	⊕ ⊕ ⊕⊝ Moderate[5]	NA	NA
	**Non-Early Cardioversionn vs Vano**	⊕ ⊕ ⊕⊝ Moderate[5]	NA	NA
	**Non-Early Cardioversion vs Vern**	⊕ ⊕ ⊕⊝ Moderate[5]	NA	NA
	**Amio vs Flec**	NA	⊕ ⊕⊝ ⊝ Low[3,4,5]	NA
	**Amio vs Prop**	NA	⊕ ⊕⊝ ⊝ Low[3,4,5]	NA
	**Amio vs Vano**	NA	⊕ ⊕⊝ ⊝ Low[3,4,5]	NA

[1]Within-study bias (ie, risk of bias in the included studies),[2]Across-study bias (ie, publication and reporting bias), [3]Indirectness, [4]Imprecision: confidence intervals include values that lead into different clinical Decisions; [5]Heterogeneity,[6]Incoherence (ie, differences between direct and indirect evidence)

Abbreviations: TIA: Transient Ischemic Attack; Amio: Amiodarone; Sota: Sotalol; Vern: Vernakalant; Vano: Vanoxerine; Prop: Propafenone; Flec: Flecainide; NA: not available.

Table S2. GRADE quality of evidence of all endpoints using CINeMA (continued).

Endpoints	Comparisons	Direct evidence<br>Quality of evidence	Indirect evidence<br>Quality of evidence	Network meta-analysis<br>Quality of evidence
**Hypotension**	**Amio vs Vern**	NA	⊕⊕⊖⊖ Low[4,5]	NA
	**Flec vs Prop**	NA	⊕⊕⊖⊖ Low[4,5]	NA
	**Flec vs Sota**	NA	⊕⊕⊖⊖ Low[4,5]	NA
	**Flec vs Vano**	NA	⊕⊕⊖⊖ Low[4,5]	NA
	**Flec vs Vern**	NA	⊕⊕⊖⊖ Low[4,5]	NA
	**Prop vs Sota**	NA	⊕⊕⊖⊖ Low[4,5]	NA
	**Prop vs Vano**	NA	⊕⊕⊖⊖ Low[4,5]	NA
	**Prop vs Vern**	NA	⊕⊕⊖⊖ Low[4,5]	NA
	**Sota vs Vano**	NA	⊕⊕⊖⊖ Low[4,5]	NA
	**Sota vs Vern**	NA	⊕⊕⊖⊖ Low[4,5]	NA
	**Vano vs Vern**	NA	⊕⊕⊖⊖ Low[4,5]	NA
**Gastrointestinal Disorders**	**Amio vs Flec**	⊕⊕⊕⊖ Moderate[5]	⊕⊕⊖⊖ Low[4,5]	⊕⊕⊖⊖ Low[4,5,6]
	**Amio vs Non-Early Cardioversion**	⊕⊕⊕⊖ Moderate[5]	⊕⊕⊖⊖ Low[4,5]	⊕⊕⊖⊖ Low[4,5,6]
	**Amio vs Prop**	⊕⊕⊕⊖ Moderate[5]	⊕⊕⊖⊖ Low[4,5]	⊕⊕⊖⊖ Low[4,5,6]
	**Amio vs Sota**	⊕⊕⊕⊖ Moderate[5]	⊕⊕⊖⊖ Low[4,5]	⊕⊕⊖⊖ Low[4,5,6]
	**Flec vs Non-Early Cardioversion**	⊕⊕⊕⊖ Moderate[5]	⊕⊕⊖⊖ Low[4,5]	⊕⊕⊖⊖ Low[4,5,6]
	**Flec vs Prop**	⊕⊕⊕⊖ Moderate[5]	⊕⊕⊖⊖ Low[4,5]	⊕⊕⊖⊖ Low[4,5,6]
	**Non-Early Cardioversion vs Prop**	⊕⊕⊕⊖ Moderate[5]	⊕⊕⊖⊖ Low[4,5]	⊕⊕⊖⊖ Low[4,5,6]
	**Non-Early Cardioversion vs Sota**	⊕⊕⊕⊖ Moderate[5]	⊕⊕⊖⊖ Low[4,5]	⊕⊕⊖⊖ Low[4,5,6]
	**Non-Early Cardioversion vs Vano**	⊕⊕⊕⊖ Moderate[5]	NA	NA
	**Non-Early Cardioversion vs Vern**	⊕⊕⊕⊖ Moderate[5]	NA	NA

	**Amio vs Vano**	NA	⊕⊕⊝⊝ Low[4,5]	NA
	**Amio vs Vern**	NA	⊕⊕⊝⊝ Low[4,5]	NA
	**Flec vs Sota**	NA	⊕⊕⊝⊝ Low[4,5]	NA
	**Flec vs Vano**	NA	⊕⊕⊝⊝ Low[4,5]	NA
	**Flec vs Vern**	NA	⊕⊕⊝⊝ Low[4,5]	NA
	**Prop vs Sota**	NA	⊕⊕⊝⊝ Low[4,5]	NA
	**Prop vs Vano**	NA	⊕⊕⊝⊝ Low[4,5]	NA
	**Prop vs Vern**	NA	⊕⊕⊝⊝ Low[4,5]	NA
	**Sota vs Vano**	NA	⊕⊕⊝⊝ Low[4,5]	NA
	**Sota vs Vern**	NA	⊕⊕⊝⊝ Low[4,5]	NA
	**Vano vs Vern**	NA	⊕⊕⊝⊝ Low[4,5]	NA
**Nervous System Disorders**	**Amio vs Non-Early Cardioversion**	⊕⊕⊕⊝ Moderate[5]	⊕⊕⊝⊝ Low[3,5]	⊕⊕⊝⊝ Low[3,5,6]
	**Amio vs Sota**	⊕⊕⊕⊝ Moderate[5]	⊕⊕⊝⊝ Low[3,5]	⊕⊕⊝⊝ Low[3,5,6]
	**Non-Early Cardioversion vs Sota**	⊕⊕⊕⊝ Moderate[5]	⊕⊕⊝⊝ Low[3,5]	⊕⊕⊝⊝ Low[3,5,6]
	**Non-Early Cardioversion vs Prop**	⊕⊕⊕⊝ Moderate[5]	NA	NA
	**Flec vs Non-Early Cardioversion**	⊕⊕⊕⊝ Moderate[5]	NA	NA
	**Non-Early Cardioversion vs Vano**	⊕⊕⊕⊝ Moderate[5]	NA	NA
	**Non-Early Cardioversion vs Vern**	⊕⊕⊕⊝ Moderate[5]	NA	NA
	**Amio vs Flec**	NA	⊕⊕⊝⊝ Low[4,5]	NA
	**Amio vs Prop**	NA	⊕⊕⊝⊝ Low[4,5]	NA
	**Amio vs Vano**	NA	⊕⊕⊝⊝ Low[4,5]	NA
	**Amio vs Vern**	NA	⊕⊕⊝⊝ Low[4,5]	NA
	**Flec vs Prop**	NA	⊕⊕⊝⊝ Low[4,5]	NA

**Flec vs Sota**	NA	⊕⊕⊖⊖ Low[4,5]	NA
**Flec vs Vano**	NA	⊕⊕⊖⊖ Low[4,5]	NA
**Flec vs Vern**	NA	⊕⊕⊖⊖ Low[4,5]	NA
**Prop vs Sota**	NA	⊕⊕⊖⊖ Low[4,5]	NA
**Prop vs Vano**	NA	⊕⊕⊖⊖ Low[4,5]	NA
**Prop vs Vern**	NA	⊕⊕⊖⊖ Low[4,5]	NA
**Sota vs Vano**	NA	⊕⊕⊖⊖ Low[4,5]	NA

[1]Within-study bias (ie, risk of bias in the included studies),[2]Across-study bias (ie, publication and reporting bias), [3]Indirectness, [4]Imprecision: confidence intervals include values that lead into different clinical Decisions; [5]Heterogeneity,[6]Incoherence (ie, differences between direct and indirect evidence)

Abbreviations: TIA: Transient Ischemic Attack; Amio: Amiodarone; Sota: Sotalol; Vern: Vernakalant; Vano: Vanoxerine; Prop: Propafenone; Flec: Flecainide; NA: not available.

Table S2. GRADE quality of evidence of all endpoints using CINeMA (continued).

Endpoints	Comparisons	Direct evidence Quality of evidence	Indirect evidence Quality of evidence	Network meta-analysis Quality of evidence
**Nervous system Disorders**	**Sota vs Vern**	NA	⊕⊕⊝⊝ Low[2,5]	NA
	**Vano vs Vern**	NA	⊕⊕⊝⊝ Low[2,5]	NA
**All caused Mortality**	**Non-Early Cardioversion vs Vern**	⊕⊕⊝⊝ Low[2,5]	NA	NA
**Stroke or TIA**	**Amio vs Non-Early Cardioversion**	⊕⊕⊝⊝ Low[2,5]	⊕⊝⊝⊝ Very Low[2,4,5,6]	⊕⊝⊝⊝ Very Low[2,4,5,6]
	**Amio vs Sota**	⊕⊕⊝⊝ Low[2,5]	⊕⊝⊝⊝ Very Low[2,4,5,6]	⊕⊝⊝⊝ Very Low[2,4,5,6]
	**Flec vs Non-Early Cardioversion**	⊕⊕⊝⊝ Low[2,5]	NA	NA
	**Non-Early Cardioversion vs Sota**	⊕⊕⊝⊝ Low[2,5]	⊕⊝⊝⊝ Very Low[2,4,5,6]	⊕⊝⊝⊝ Very Low[2,4,5,6]
	**Non-Early Cardioversion vs Vern**	⊕⊕⊝⊝ Low[2,5]	NA	NA
	**Amio vs Flec**	NA	⊕⊝⊝⊝ Very Low[2,4,5,6]	NA
	**Amio vs Vern**	NA	⊕⊝⊝⊝ Very Low[2,4,5,6]	NA
	**Flec vs Sota**	NA	⊕⊝⊝⊝ Very Low[2,4,5,6]	NA
	**Flec vs Vern**	NA	⊕⊝⊝⊝ Very Low[2,4,5,6]	NA
	**Sota vs Vern**	NA	⊕⊝⊝⊝ Very Low[2,4,5,6]	NA
**Heart Failure**	**Amio vs Non-Early Cardioversion**	⊕⊕⊕⊝ Moderate[5]	⊕⊝⊝⊝ Low[2,5]	⊕⊕⊝⊝ Low[2,5,6]
	**Amio vs Sota**	⊕⊕⊕⊝ Moderate[5]	⊕⊝⊝⊝ Low[2,5]	⊕⊕⊝⊝ Low[2,5,6]
	**Non-Early Cardioversion vs Sota**	⊕⊕⊕⊝ Moderate[5]	⊕⊝⊝⊝ Low[2,5]	⊕⊕⊝⊝ Low[2,5,6]
	**Non-Early Cardioversion vs Vern**	⊕⊕⊕⊝ Moderate[5]	NA	NA
	**Amio vs Vern**	NA	⊕⊕⊝⊝ Low[2,5]	NA
	**Sota vs Vern**	NA	⊕⊕⊝⊝ Low[2,5]	NA

[1]Within-study bias (ie, risk of bias in the included studies),[2]Across-study bias (ie, publication and reporting bias), [3]Indirectness, [4]Imprecision: confidence intervals include values that lead into different clinical

Decisions; [5]Heterogeneity,[6]Incoherence (ie, differences between direct and indirect evidence)

Abbreviations: TIA: Transient Ischemic Attack; Amio: Amiodarone; Sota: Sotalol; Vern: Vernakalant; Vano: Vanoxerine; Prop: Propafenone; Flec: Flecainide; NA: not available.

Supplemental Table 3. Assessment of heterogeneity and consistency, for the safety endpoints among different antiarrhythmic drugs as early pharmacologic cardioversion.

Endpoints	Comparison	Indirect			Direct			Network			Heterogeneity	Consistency	
		OR	LL	UL	OR	LL	UL	OR	LL	UL	τ^2	Global P>X [2]	Local P>z
Cardioversion to sinus rhythm within 24h	Amio vs Non-Early Cardioversion	3.12	1.04	9.10	3.16	1.15	9.46	3.14	1.13	8.77	0.802		
	Flec vs Non-Early Cardioversion	9.86	2.02	41.08	10.01	2.02	50.55	10.04	1.99	50.57	0.780		
	Prop vs Non-Early Cardioversion	14.48	3.56	36.60	12.18	3.67	40.45	11.32	3.75	34.16	0.669		
	Sota vs Non-Early Cardioversion	4.95	0.64	36.60	1.82	0.30	11.02	1.78	0.37	8.69	0.762	0.795	0.835
	Flec vs Amio	3.32	1.55	20.09	1.25	1.08	18.17	2.46	1.57	9.97	0.032		
	Prop vs Amio	3.32	2.30	14.88	2.00	1.07	13.50	2.53	1.71	9.03	0.532		
	Amio vs Sota	1.43	1.06	3.32	2.13	1.20	6.69	1.75	1.20	2.72	0.297		
	Prop vs Flec	1.16	0.17	7.39	0.79	0.06	11.02	1.03	0.22	4.48	0		
Maintaining in sinus rhythm within 1-week	Sota vs Non-Early Cardioversion	5.47	1.21	329.45	5.47	1.06	492.75	5.00	1.27	383.47	0		
	Amio vs Non-Early Cardioversion	0.41	0.00	244.69	0.41	0.00	36.60	2.38	0.03	175.92	0	0.897	0.946
	Sota vs Amio	2.18	1.02	133.94	2.20	1.02	200.33	2.18	1.07	66.69	0		
Bradycardia	Sota vs Non-Early Cardioversion	1.40	0.01	148.41	1.92	0.03	148.41	1.70	0.15	19.08	0.453		
	Amio vs Non-Early Cardioversion	0.38	0.00	73.70	0.54	0.02	9.03	1.23	0.23	6.74	0	0.530	0.604
	Amio vs Sota	1.09	0.01	181.27	0.73	0.01	20.09	0.86	0.04	12.18	0.192		
Hypotension	Sota vs Non-Early Cardioversion	2.46	0.02	403.43	5.99	0.65	473.18	5.17	0.22	108.84	0		
	Amio vs Non-Early Cardioversion	0.13	0.01	1.51	0.20	0.01	4.06	1.57	0.43	5.75	0.702	0.653	0.630
	Amio vs Sota	0.98	0.07	117.16	0.51	0.01	20.09	0.52	0.04	29.96	0.578		
Gastrointestinal disorders	Amio vs Non-Early Cardioversion	1.09	0.00	215.14	1.03	0.01	148.41	0.99	0.22	12.14	0.322		
	Flec vs Non-Early Cardioversion	0.23	0.00	4.91	0.09	0.00	5.47	2.04	0.11	38.46	0.000		
	Prop vs Non-Early Cardioversion	9.36	1.99	5.47	24.15	4.48	420.25	22.21	1.13	595.14	0.198		
	Sota vs Non-Early Cardioversion	NA	NA	NA	NA	0.99	NA	NA	NA	NA	NA	0.555	0.558
	Prop vs Amio	66.69	0.10	59874.14	97.45	1.95	68421.54	164.02	0.93	162754.79	0.303		
	Flec vs Amio	36.60	0.05	59874.14	83.27	0.02	70220.35	33.12	0.04	59874.14	0.599		
	Sota vs Amio	0.00	0.23	0.99	0.00	0.24	0.99	0.00	0.27	0.99	0.943		
	Flec vs Prop	0.49	0.03	107.13	0.34	0.99	102.21	0.20	0.00	49.40	0.266		
Nervous system disorders	Sota vs Non-Early Cardioversion	NA	NA	NA	NA	0.99	NA	NA	NA	NA	NA		
	Amio vs Non-Early Cardioversion	8.32	0.99	303.36	2.72	0.99	171.15	2.53	0.10	63.80	0	0.451	1
	Amio vs Sota	0.00	0.98	196.11	0.27	0.00	1011.12	0.01	0.00	148.41	0		
Stroke or TIA	Sota vs Non-Early Cardioversion	0.71	0.01	9.90	0.41	0.03	3.67	0.30	0.03	2.96	0.675		
	Amio vs Non-Early Cardioversion	0.08	0.01	1.04	0.13	0.02	2.15	0.28	0.03	2.79	0.906	0	0
	Sota vs Amio	0.00	0.03	1784.82	0.01	0.01	6566.00	0.00	0.03	8955.29	0.883		
Heart failure	Sota vs Non-Early Cardioversion	0.01	0.00	13.46	0.06	0.00	29.96	0.06	0.00	26.10	0.964		
	Amio vs Non-Early Cardioversion	0.98	0.07	18.63	0.92	0.02	18.17	0.79	0.06	11.14	0	0.210	0.380
	Amio vs Sota	1.54	0.99	29.96	1.19	0.99	24.53	1.88	0.01	54.60	0.809		

Abbreviations: TIA: Transient Ischemic Attack; Amio: Amiodarone; Sota: Sotalol; Vern: Vernakalant; Vano: Vanoxerine; Prop: Propafenone; Flec: Flecainide; NA: not available.

Supplemental Table 4. Assessment of Bayesian random-effects model fit and inconsistency, for all endpoints in the study group with early pharmacologic cardioversion arm as different antiarrhythmic drugs.

Endpoints	Model fit			Node-splitting inconsistency P-values							
	Data points	Total residual Variance*	DIC†	Amio vs Non-Early Cardioversion	Flec vs Non-Early Cardioversion	Non-Early Cardioversion vs Prop	Non-Early Cardioversion vs Sota	Amio vs Flec	Amio vs Prop	Amio vs Sota	Flec vs Prop
**Cardioversion to sinus rhythm within 24 hours**	48	47.7	92.2	0.718	0.751	0.394	0.454	0.535	0.407	0.452	0.804
**Maintaining in sinus rhythm within 1-week**	12	12.2	24.2	0.998	NA	NA	0.999	NA	NA	1.000	NA
**Bradycardia**	30	29.9	52.9	0.908	NA	NA	0.910	NA	NA	0.883	NA
**Hypotension**	24	20.9	36.7	0.153	NA	NA	0.164	NA	NA	0.161	NA
**Gastrointestinal disorders**	30	19.3	34.1	0.433	0.007	0.005	0.591	0.197	0.593	0.693	0.010
**Nervous system disorders**	14	9.2	18.2	0.645	NA	NA	0.618	NA	NA	0.789	NA
**Stroke or TIA**	18	9.5	18.6	0.795	NA	NA	0.917	NA	NA	0.874	NA
**Heart failure**	14	9.2	16.9	0.936	NA	NA	0.953	NA	NA	0.908	NA

*Once total residual variance approximated the number of data points, it means a good model fit; †DIC refers to deviance information criterion, lower values of DIC are better.

Abbreviations: TIA: Transient Ischemic Attack; Amio: Amiodarone; Sota: Sotalol; Vern: Vernakalant; Vano: Vanoxerine; Prop: Propafenone; Flec: Flecainide;DIC: Deviance Information criterion; NA: not available.
