# Supplementary material for: Effect of Early Pharmacologic Cardioversion vs. Non-early Cardioversion in the Patients With Recent-Onset Atrial Fibrillation Within 4-Week Follow-Up Period: A Systematic Review and Network Meta-Analysis
Source: Front Cardiovasc Med. 2022 Apr 11;9:843939. doi: 10.3389/fcvm.2022.843939 (PMC9036487; doi:10.3389/fcvm.2022.843939)
Supplement: Supplementary file 2 [file Data_Sheet_2.doc]

**The PRISMA for NMA Checklist of Items to Include When Reporting A Systematic Review Involving a Network Meta-analysis**

| **TITLE** | **CHECKLIST ITEM** | **REPORTED ON PAGE #** |
| --- | --- | --- |
| 1. Title: | Identify the report as a systematic review *incorporating a network meta-analysis (or related form of meta-analysis).* | #1 |
| **ABSTRACT** |  |  |
| 2.Objective | A clear statement of the main aim of the study and the major hypothesis tested or research question posed | #2 |
| 3.Design | Including factors such as prospective, randomisation, blinding, placebo control, case control, crossover, criterion standards for diagnostic tests, etc. | #2 |
| 4.Information sources | Key databases searched and search dates. | #2 |
| 5.Methods | This should normally include study eligibility criteria, study appraisal and synthesis methods. | #2 |
| 6.Results | Main results with (for quantitative studies) 95% confidence intervals and, where appropriate, the exact level of statistical significance and the number need to treat/harm. Whenever possible, state absolute rather than relative risks. | #2 |
| 7.Conclusions | Primary conclusions and their implications, suggesting areas for further research if appropriate. Do not go beyond the data in the article. Conclusions are important because this is often the only part that readers look at. | #2 |
| 8.Trial registration | Registry and number (for clinical trials and, if available, for observational studies and systematic reviews). | #2 |
| **INTRODUCTION** |  |  |
| 9.Rationale | Describe the rationale for the review in the context of what is already known*, including mention of why a network meta-analysis has been conducted.* | #3 |
| 10. Objectives: | The research question including components such as participants, interventions, comparators, and outcomes. | #3 |
| **METHODS** |  |  |
| 11.Protocol and registration | Indicate whether a review protocol exists and if and where it can be accessed (e.g., Web address); and, if available, provide registration information, including registration number. | #3 |
| 12. Eligibility criteria: | Study and report characteristics used as criteria for inclusion. | #3,#4 |
| 13. Information sources: | Key databases searched and search dates. | #3,#4 |
| 14.Search | Present full electronic search strategy for at least one database, including any limits used, such that it could be repeated. | #3,#4 |
| 15.Study selection | State the process for selecting studies (i.e., screening, eligibility, included in systematic review, and, if applicable, included in the meta-analysis). | #3,#4 |
| 16.Data collection process | Describe method of data extraction from reports (e.g., piloted forms, independently, in duplicate) and any processes for obtaining and confirming data from investigators. | #3,#4,#5 |
| 17.Data items | List and define all variables for which data were sought (e.g., PICOS, funding sources) and any assumptions and simplifications made. | #3,#4 |
| 1. Risk of bias within individual studies | Describe methods used for assessing risk of bias of individual studies (including specification of whether this was done at the study or outcome level), and how this information is to be used in any data synthesis. | #4,#5 |
| 19.Geometry of the network | Describe methods used to explore the geometry of the treatment network under study and potential biases related to it. This should include how the evidence base has been graphically summarized for presentation, and what characteristics were compiled and used to describe the evidence base to readers. | #4,#5 |
| 20.Summary measures | State the principal summary measures (e.g., risk ratio, difference in means). *Also describe the use of additional summary measures assessed, such as treatment rankings and surface under the cumulative ranking curve (SUCRA) values, as well as modified approaches used to present summary findings from meta-analyses.* | #4,#5 |
| 21.Planned methods of analysis | Describe the methods of handling data and combining results of studies for each network meta-analysis. This should include, but not be limited to:   - *Handling of multi-arm trials;* - *Selection of variance structure;* - *Selection of prior distributions in Bayesian analyses; and*   *Assessment of model fit.* | #4,#5 |
| 22.Assessment of Inconsistency | Describe the statistical methods used to evaluate the agreement of direct and indirect evidence in the treatment network(s) studied. Describe efforts taken to address its presence when found. | #4,#5 |
| 23.Risk of bias across studies | Specify any assessment of risk of bias that may affect the cumulative evidence (e.g., publication bias, selective reporting within studies). | #4,#5 |
| 24.Additional analyses | Describe methods of additional analyses if done, indicating which were pre-specified. This may include, but not be limited to, the following:   - Sensitivity or subgroup analyses; - Meta-regression analyses; - *Alternative formulations of the treatment network; and*   *Use of alternative prior distributions for Bayesian analyses (if applicable).* | #4,#5 |
| **RESULTS†** |  |  |
| 25. Included studies: | Number and type of included studies and participants and relevant characteristics of studies. | #5, #6 |
| 26.Presentation of network structure | Provide a network graph of the included studies to enable visualization of the geometry of the treatment network. | #5, #6, #7, #8 |
| 27.Summary of network geometry | Provide a brief overview of characteristics of the treatment network. This may include commentary on the abundance of trials and randomized patients for the different interventions and pairwise comparisons in the network, gaps of evidence in the treatment network, and potential biases reflected by the network structure. | #5, #6, #7, #8 |
| 28.Study characteristics | For each study, present characteristics for which data were extracted (e.g., study size, PICOS, follow-up period) and provide the citations. | #5, #6, #7 |
| 29.Risk of bias within studies | Present data on risk of bias of each study and, if available, any outcome level assessment. | #6, #7, #8 |
| 30.Synthesis of results | For all outcomes considered (benefits or harms), present, for each study: 1) simple summary data for each intervention group, and 2) effect estimates and confidence intervals. *Modified approaches may be needed to deal with information from larger networks.* | #6, #7, #8 |
| 31.Exploration for inconsistency | Describe results from investigations of inconsistency. This may include such information as measures of model fit to compare consistency and inconsistency models, *P* values from statistical tests, or summary of inconsistency estimates from different parts of the treatment network. | #6, #7,#8 |
| 32.Risk of bias across studies | Present results of any assessment of risk of bias across studies for the evidence base being studied. | #6, #7,#8 |
| 33.Results of additional analyses | Give results of additional analyses, if done (e.g., sensitivity or subgroup analyses, meta-regression analyses*, alternative network geometries studied, alternative choice of prior distributions for Bayesian analyses,* and so forth). | #7,#8 |
| **DISCUSSION** |  |  |
| 34. Interpretation: | General interpretation of the results and important implications | #9,#10,#11 |
| 35. Strengths and Limitations of evidence: | Brief summary of strengths and limitations of evidence (e.g. inconsistency, imprecision, indirectness, or risk of bias, other supporting or conflicting evidence) | #9,#10,#11 |
| 36. Description of the effect: | Direction of the effect (i.e. which group is favoured) and size of the effect in terms meaningful to clinicians and patients. | #9,#10,#11 |
| 37.Conclusions | Provide a general interpretation of the results in the context of other evidence, and implications for future research. | #11 |
| **OTHER** |  |  |
| 38. Funding: | Primary source of funding for the review. | #12 |
|  |  |  |
|  |  |  |
|  |  |  |

PICOS = population, intervention, comparators, outcomes, study design.

† Authors may wish to plan for use of appendices to present all relevant information in full detail for items in this section.
